# Incorporating the type and direction information in predicting novel regulatory interactions between HIV-1 and human proteins using a biclustering approach

**DOI:** 10.1186/1471-2105-15-26

**Published:** 2014-01-24

**Authors:** Anirban Mukhopadhyay, Sumanta Ray, Ujjwal Maulik

**Affiliations:** 1 Department of Computer Science and Engineering, University of Kalyani, Kalyani-741235, West Bengal, India; 2 Department of Computer Science and Engineering, Aliah University, Kolkata-700091, West Bengal, India; 3 Department of Computer Science and Engineering, Jadavpur University, Kolkata-700032, West Bengal, India

## Abstract

**Background:**

Discovering novel interactions between HIV-1 and human proteins would greatly contribute to different areas of HIV research. Identification of such interactions leads to a greater insight into drug target prediction. Some recent studies have been conducted for computational prediction of new interactions based on the experimentally validated information stored in a HIV-1-human protein-protein interaction database. However, these techniques do not predict any regulatory mechanism between HIV-1 and human proteins by considering *interaction types* and *direction of regulation* of interactions.

**Results:**

Here we present an association rule mining technique based on biclustering for discovering a set of rules among human and HIV-1 proteins using the publicly available HIV-1-human PPI database. These rules are subsequently utilized to predict some novel interactions among HIV-1 and human proteins. For prediction purpose both the *interaction types* and *direction of regulation* of interactions, (i.e., virus-to-host or host-to-virus) are considered here to provide important additional information about the regulation pattern of interactions. We have also studied the biclusters and analyzed the significant GO terms and KEGG pathways in which the human proteins of the biclusters participate. Moreover the predicted rules have also been analyzed to discover regulatory relationship between some human proteins in course of HIV-1 infection. Some experimental evidences of our predicted interactions have been found by searching the recent literatures in PUBMED. We have also highlighted some human proteins that are likely to act against the HIV-1 attack.

**Conclusions:**

We pose the problem of identifying new regulatory interactions between HIV-1 and human proteins based on the existing PPI database as an association rule mining problem based on biclustering algorithm. We discover some novel regulatory interactions between HIV-1 and human proteins. Significant number of predicted interactions has been found to be supported by recent literature.

## Background

Human immunodeficiency virus-1 (HIV-1) causes acquired immunodeficiency syndrome (AIDS) in which human immune system begins to collapse. Progressive failure of the immune system leads to life threatening infection. At each stage of life cycle, HIV-1 virus hijacks the host cellular machinery for increasing the production of virus genomic material. HIV-1 virus contains a single stranded RNA genome, which codes for only 19 proteins; thus, it relies on human cellular functions. The RNA genome, consisting of seven structural landmarks (LTR, TAR, RRE, PE, SLIP, CRS, and INS) and nine genes (gag, pol, env, tat, rev, nef, vif, vpr, and vpu), encode nineteen proteins. The prediction of possible viral-host interactions is one of the major tasks in Protein-Protein Interaction (PPI) research for antiviral drug discovery and treatment optimization. Predicting PPIs between viral and host proteins has contributed substantial knowledge to the drug design area. Recently, PPI prediction has been regarded as an promising alternative to the traditional approach to drug design [1]. Novel predictions can provide sound knowledge to the drug developers for understanding the mechanism of infection and assisting them to accelerate the development of new therapeutic approaches.

The computational approaches for predicting PPIs are mainly modeled as classification problems [2]. In [3] a Bayesian classification based approach is proposed for predicting PPIs in yeast. An assessment based on the genomic features used in a Bayesian network approach to predict genome-wide PPIs in yeast is proposed in [4]. Using a variant of kernel canonical correlation analysis the pathway protein interactions have been predicted in [5]. Afterwards an approach called Mixture-of-Feature-Experts (mixture of classifiers) [6], some kernel based methods [7] and a decision tree based method [8] have been constructed to predict the set of interacting proteins in yeast and human cells.

Most of the approaches were primarily focused to determine the PPIs in a single organism ("intra-species prediction"). But the prediction of PPIs between different organisms ("inter-species prediction"), more specifically in virus and the corresponding host proteins is now very important issue in development of new therapeutic approaches and design of drugs for these viral diseases. Recently some computational approaches are proposed by several researchers to predict and analyze some novel interactions between HIV-1 and human proteins.

In [9] a random forest classifier model is utilized for predicting new HIV-1-human PPIs. The authors extended their method by integrating a semi-supervised approach for including partial positive interactions in [10]. A structural similarity based approach for predicting HIV-1-human protein interactions is proposed in [11]. A support vector machine classifier based approach is presented in [12]. Recently a biclustering technique is used to identify significant host-cellular subsystem in [13]. They found significant patterns of HIV-host interaction in order to identify core processes that are active during infection. They have used a distance measure to group the host protein sets and identified 37 distinct higher-level subsystems and highlighted significant host-cell subsystems that are perturbed during the course of HIV-1 infection. The interaction types between the proteins are considered but the direction of regulation of these interactions are not focused here.

A similar biclustering approach is studied in [14] to find immunodeficiency gateway proteins and their involvement in microRNA regulation. The authors make an exhaustive graph search technique to identify the strong significant biclusters from the HIV-1-human protein interaction network, modeled as a bipartite graph. These strong significant biclusters or bicliques are then analyzed to find out the activity of miRNAs through the HIV-1 regulatory pathway in human at systems level.

In another study [15], a novel association rule mining approach based on biclustering is proposed for finding frequent closed itemsets [16] followed by a set of association rules from the adjacency matrix of the HIV-1–human interaction network. These rules are then utilized for predicting new interactions. In both studies [14,15] the interaction types and regulation direction of the HIV-1 proteins and human proteins are not considered for finding the bicliques.

With this observation we use an association rule mining approach for finding a set of rules by considering both the interaction types and the direction of regulations. For this, we have annotated each interaction with interaction type and divided the whole network into two annotated subnetworks depending on the regulation direction of interaction type. We have utilized Binary inclusion-Maximal (BiMax) biclustering algorithm [17] on each subnetwork and identified all maximal biclusters from these two matrices. We have considered the biclusters found from each subnetwork separately, and generated all possible association rules satisfying the minimum support and minimum confidence thresholds. Subsequently some interactions between HIV-1 proteins and human proteins are predicted using those association rules. As information about the direction of regulation and types of interactions are already embedded in the association rules, the predicted interactions from those rules also inherit those information. These additional information about the predicted interactions may contribute substantial knowledge in understanding HIV pathogenesis.

## Method

**Figure 1:**
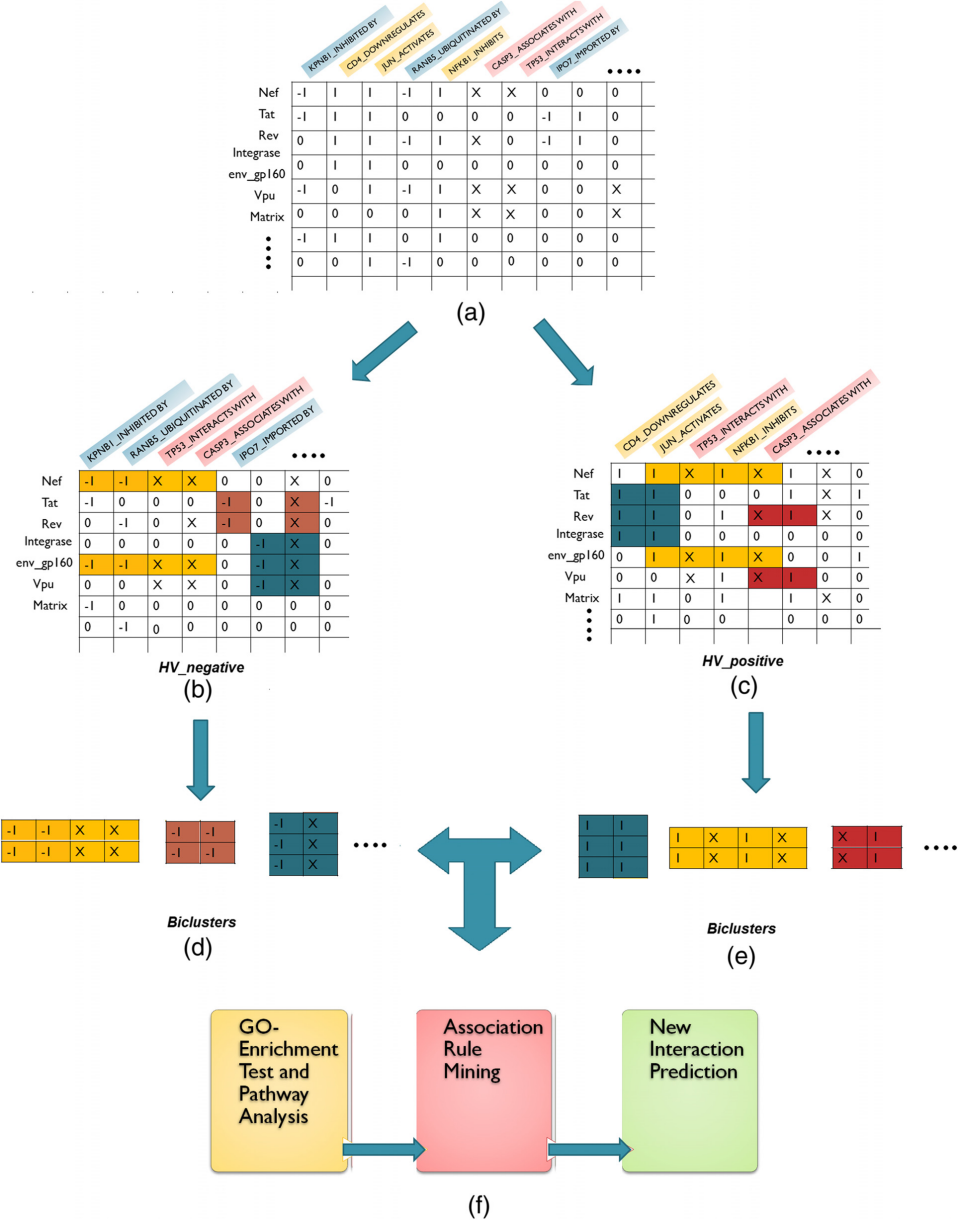
**This Figure summarizes the whole process.** In the first step the whole bipartite network is broadly partitioned in two networks based on the three classes of interaction types (shown in panels **a–c**). Then biclustering is performed on each network to get significant bicliques (shown in panels **d–e**). In the third step these bicliques are analyzed and some association rules are extracted from those biclusters. After that some novel interactions are predicted (shown in panel **f**).

In this section biclustering-based association rule mining approach is described. An outline of our method for analysis of bicliques and prediction of interactions has been visualized in Figure 1.

### Preparation of the HIV-1-human PPI Bipartite Network

**Figure 2:**
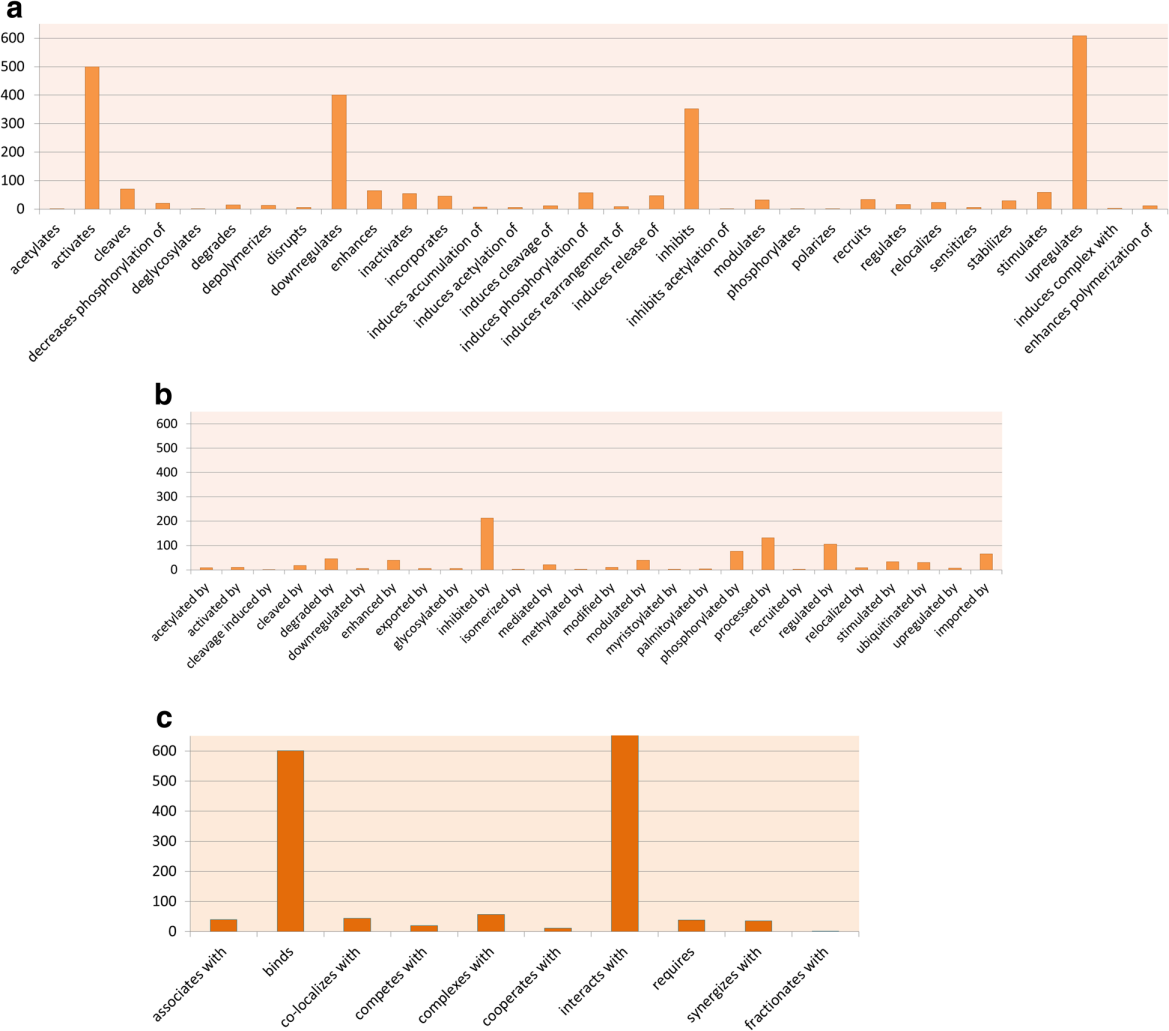
**Bar diagram showing the distribution of interaction types in the whole HHPID dataset.** Panel-**a**, Panel-**b** and Panel-**c** show distribution of edges annotated by class-1, class-2 and class-3 type interactions, respectively.

The HIV-1-human PPI dataset which is published in [18] consists of total 5127 interactions between 19 HIV-1 proteins and 1432 human proteins. For each interactions there is an associated interaction type. We broadly divide all the interaction types in three classes: regulating, regulated by and bidirectional (regulation is in both way). We find 68 unique interaction types (among them 33 are in class 1, 25 are in class 2 and the remaining 10 are in class 3) that are listed in Table 1. We draw a bar diagram shown in Figure 2 that shows the distribution of interaction types in the HHPID dataset. Figures 2(a), (b) and (c) represent the distribution of the edges annotated with corresponding interaction types spanned in three classes respectively. By annotating each human protein with its corresponding interaction type we get 2564 annotated human proteins considering the two classes (regulating and bidirectional) of interactions and 1271 annotated human proteins considering the other two classes (regulated by and bidirectional) of interaction types. For example, a protein of type *HP*1_*upregulates* signifies that the HP1 protein is upregulated by some viral proteins and protein of type *HP*2_*inhibitedby* represents that the human protein HP2 inhibits some viral protein. We construct two binary matrices of human and viral proteins, *HV*_ *positive* of size 19 × 2564, and *HV*_*negative* of size 19 × 1271. An entry of ‘1’ in matrix *HP*_ *positive* and ‘-1’ in *HV*_*negative* denotes the presence of interaction between the corresponding pair of human and HIV-1 proteins, and an entry of 0 represents the absence of any information regarding the interaction of the corresponding human and viral proteins. An entry ‘X’ in both the matrices represents the interaction between the corresponding pair of human and HIV-1 proteins is two-way or bidirectional interaction. The whole process is described in detail in algorithm 1. The step-1 and step-2 are involved in preparing the datasets in the form of two matrices. In step-3 and step-4 our algorithm uses BiMax [17] as a subroutine for finding the maximal frequent closed itemsets or biclusters with respect to a *minsupport* value and *minconfidence* value specified in the algorithm.

**Table 1:** The three classes of interactions and corresponding interaction types

**Interaction classes**	**Interaction types**
Class-1 (Direction of regulation is from viral to host proteins)	acetylates, activates, cleaves, decreases phosphorylation of, deglycosylates, degrades,depolymerizes, disrupts, downregulates, enhances, enhances polymerization of,inactivates, incorporates, induces accumulation of, induces acetylation of, inducescleavage of, induces complex with, induces phosphorylation of, induces rearrangement of, induces release of, inhibits, inhibits acetylation of, modulates, phosphorylates, polarizes, recruits, upregulates, relocalizes, sensitizes, stabilizes, stimulates, upregulates, regulates
Class-2 (Direction of regulation is from host to viral proteins)	acetylated by, activated by, cleavage induced by, cleaved by, degraded by,downregulated by, enhanced by, exported by, glycosylated by, imported by, inhibited by, isomerized by, mediated by, methylated by, modified by, modulated by, myristoylated by, palmitoylated by, phosphorylated by, processed by, recruited by, regulated by,relocalized by, stimulated by, ubiquitinated by, upregulated by
Class-3 (Bidirectional)	co-localizes with, binds, competes with, complexes with, cooperates with, fractionateswith, associates with, interacts with, requires, synergizes with

Algorithm 1 Algorithm of the whole procedure

**Algorithm 2 The** ***Gen_Network* procedure**

### Finding association rules

In data mining, association rule mining (ARM) is a popular and well researched method for discovering interesting relations between variables and showing attribute-value associations that occur frequently in large databases. The problem of association rule mining is defined as follows: Let *I* = {*i*_1_,*i*_2_,…*i*_*n*_} be a set of *n* items and *X* be an itemset where *X* ⊂*I*. Let *T* = {(*t*_1_,*X*_1_),(*t*_2_,*X*_2_),…(*t*_*m*_,*X*_*m*_)} be a set of *m* transactions, where *t*_*i*_ and *X*_*i*_, *i* = 1,2,…,*m* are the transaction identifier and the associated itemset respectively. The support of an itemset *X* is the number of transactions where all the items in *X* appear. An itemset is called frequent if its support is greater than some threshold *min*_*sup*. The confidence of an Association Rule (AR) of the form *P* ⇒ *Q*, *P* ⋂ *Q* = *ϕ*, *P* ⋃ *Q* = *X* obtained from an itemset *X* is defined as the ratio of the support of *X* to the support of *P*. Formally the ARM problem can be defined as follows: find the set of all rules *R* of the form *P* ⇒ *Q* such that *P* ⋃ *Q* is a frequent itemset and the confidence of *P* ⇒ *Q* is greater than a threshold *min*_*conf*.

The concept of frequent closed itemsets [16], which are condensed representations of all frequent itemsets, is defined to avoid redundancy. An itemset is called closed itemset if none of its proper supersets have the same support value. Finding the set of frequent itemsets is equivalent to find a set of all-1 biclusters each having at least min_sup number of rows [15]. BiMax generates all maximal biclusters and the columns of each maximal bicluster represents a closed itemset. Hence all extracted biclusters satisfying min_sup condition provide the set of frequent closed itemsets.

Here the rows of the binary matrices *HV*_ *positive*, and *HV*_*negative* represent the viral proteins and the columns represent the annotated human proteins. Each row (viral protein) has been considered as a transaction and each column (human protein) represents an item. Now an item is purchased by a transaction if the corresponding value in the matrix is ‘1’ or ‘X’ or ‘-1’. This can be interpreted as follows: with a viral protein some of the human proteins are associated with specific type of interactions. Now finding the frequent closed itemsets from these two matrices is equivalent to identify the maximal all-1 biclusters with a given *min*_*sup* value representing the number of rows of these biclusters. Here BiMax algorithm is utilized for finding the maximal biclusters from these two binary matrices. These biclusters are treated as maximal frequent closed itemset for finding the association rules. Details of the method describing association rule mining that utilizes the biclustering technique are given in the Additional file 1.

Here the rules may be of types: Type-1:

$$\begin{array}{l} \left[\text{HP}1\_\text{upregulates},\text{HP}2\_\text{downregulates},\text{HP}3\_\text{activates}\right]\\ \;\Rightarrow [\{\text{HP}4,\text{HP}5\}\_\text{activates},\text{HP}6\_\text{downregulates}] \end{array}$$

and Type-2:

[VP1,VP2,VP3]⇒[VP4,VP5].

The type-1 rules may be interpreted as follows: if the human protein HP1 is upregulated, HP2 is downregulated and HP3 is activated by some set of viral proteins then there is a high chance of activation of the two proteins HP4 and HP5 and downregulation of the protein HP6 by the same set of viral proteins. The type-2 rules are interpreted as: if the viral proteins VP1, VP2, and VP3 interact with some human proteins then VP4 and VP5 are also likely to interact with these human proteins.

### Predicting new interactions

From the extracted association rules we predict some novel interactions associated with interaction types, between HIV-1 and human proteins. Consider a frequent closed itemset consisting of annotated human proteins as follows: *HP*1_ *f*1, *HP*2_ *f*2, *HP*3_ *f*3, *HP*4_ *f*4, and *HP*5_ *f*5, where each *f*_*i*_ denotes the interaction type tagged with each of these human proteins. Suppose a rule constructed from those proteins is as follows:

[HP1_f1,HP2_f2,HP3_f3]⇒[HP4_f4,HP5_f5].

**Figure 3:**
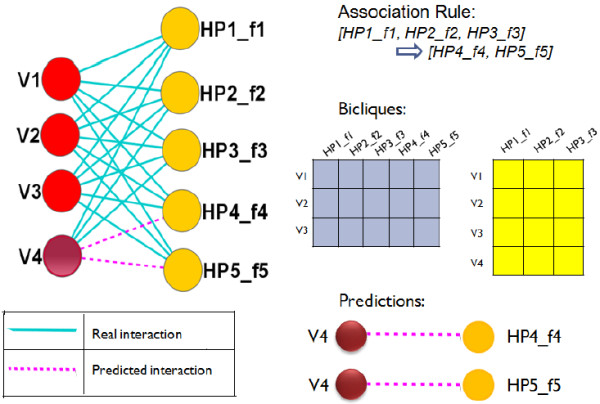
An example of prediction process from the association rules.

In this scenario we further assume that the proteins *HP*1_ *f*1, …, *HP*5_ *f*5 form a biclique with 3 viral proteins *V*1, *V*2, and *V*3 (in other words we can say that the support count for this frequent itemset is 3) shown in Figure 3. Now without loss of generality suppose the proteins in the antecedent of the rule form another biclique with 4 viral proteins: *V*1, *V*2, *V*3, and *V*4 (as the subset of a frequent itemset is always frequent, so the antecedent is true for at least 3 viral proteins). So the confidence of this rule is 3/4 or 75%. From this observation we can predict that the viral protein *V*4 is also likely to interacts with *HP*4_ *f*4 and *HP*5_ *f*5, and the confidence of this prediction is 75%. Figure 3 describes the whole scenario. This process is also applied in type-2 rules for prediction of new interactions in similar fashion.

## Results and discussion

In this section we analyze the predicted biclusters or bicliques and study the biological relevance of the human proteins constituting those bicliques. After that we show the association rules that are generated from those biclusters. For the purpose of illustrating those rules we find out biological importance of these rules that give an insight view into the regulation pattern of human proteins during the HIV-1 infection. We also show some novel predicted interactions and find out the evidences from recent literature that strengthen our prediction. For visualizing the predicted interactions we draw two bipartite graphs that include all the predictions we made here.

### Analysis of obtained bicliques

**Figure 4:**
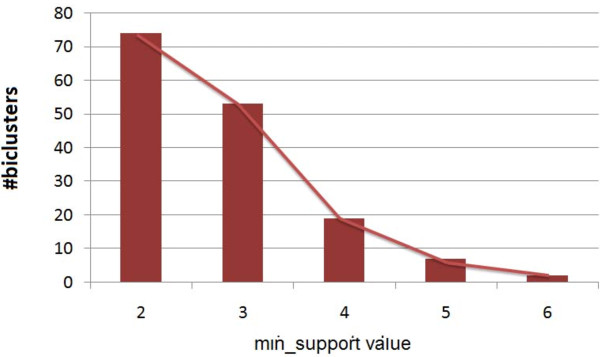
**Distribution of biclusters extracted from** ***HV*_*positive* against the** ***min*_*support* values.**

**Figure 5:**
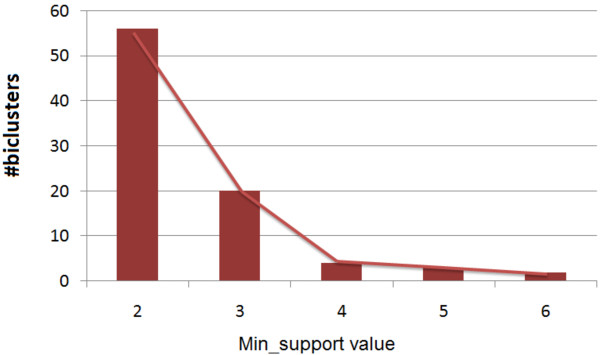
**Distribution of biclusters extracted from** ***HV*_*negative* against the** ***min*_*support* values.**

We found 19 biclusters in both of the matrices *HV*_ *positive* and *HV*_*negative*. For extracting the biclusters from *HV*_ *positive* and *HV*_*negative*, we plot the distribution of biclusters against *min*_*support* value for both *HV*_ *positive* and *HV*_*negative* matrices. From Figures 4 and 5 we notice a sharp fall of the number of biclusters when *min*_*support* value is changing from 3 to 4 for *HV*_ *positive* and the same situation is happening for *HV*_*negative* when *min*_*support* value changes from 2 to 3. To get more biologically relevant biclusters from *HV*_ *positive* matrix, we keep minimum number of viral proteins (or, *min*_*support* value) as 4 and minimum number of human proteins (or, minimum number of items) as 2, whereas in the case of *HV*_*negative* the corresponding values are 3 and 2. Thus each bicluster represents a biclique in the whole interaction graph consisting of viral proteins and human proteins as two partitioned sets of nodes. The viral and human proteins consisting these biclusters are listed in Table 2 and Table 3, respectively. Columns 4, 5 and 6 represent the most significant GO-terms, GO-ids and the corresponding p-values of three broadly classified GO category: biological process, molecular function and cellular component, respectively. We also find significant KEGG pathways for the human proteins participating in each bicluster.

**Table 2:** **The significant GO-terms, GO-id and KEGG pathways found in the bicliques extracted from** ***HV*** **_** ***positive* matrix, considering interaction types and direction of the interactions**

**Biclique**	**HIV protein**	**Human protein**	**GO term (bp)**	**GO term (cc)**	**GO term (mf)**	**KEGG pathway**
1	Tat Vpr env_gp120 matrix	BCL2 CASP3 TP53 IFNG IFNG IL10 IL2 IL6 MAPK1 NFKB1 PARP1 FOS JUN TNF	Regulation of apoptosis (GO: 0042981) (3.1E-11)	Nucleoplasm (GO:0005654) (6.8E-5)	Promoter binding (GO:0010843) (1.7E-5)	T cell receptor signaling pathway (1.2E-9)
2	Nef Tat env_gp120env_gp160	BCL2 ICAM1 IFNG IL1B IL2 IL6 MAPK1 MAPK3 FOS JUN	Positive regulation of nitrogen compound metabolic process (GO: 0051173) (1.8E-8)	Extracellular space (GO:0005615) (8.3E-4)	Cytokine activity (GO:0005125) (2.6E-4)	Toll-like receptor signaling pathway (3.3E-7)
3	Nef Vpr env_gp120env_gp160	CD4 BCL2 IFNG IL2 IL6 MAPK14 MAPK1 FOS JUN	Positive regulation of macromolecule metabolic process (GO:0010604) (2.5E-10)	Nucleoplasm (GO:0005654) (1.4E-2)	Protein dimerizationactivity (GO:0046983)(3.5E-3)	T cell receptor signaling pathway (2.2E-9)
4	Nef Tat Vpr env_gp120env_gp160	BCL2 IFNG IL2 IL6 MAPK1 FOS JUN	Positive regulation of macromolecule metabolic process (GO:0010604) (6.4E-8)	Extracellular space (GO:0005615) (3.7E-2)	Cytokine activity (GO:0005125) (3.2E-3)	T cell receptor signalingpathway (2.8E-6)
5	Tat Vpr env_gp120env_gp160	BCL2 CYCS IFNG IL2 IL6 MAPK1 FOS JUN	Regulation of apoptosis (GO:0042981) (2.9E-7)	Protein phosphatase type 2A complex (GO:0000159) (1.1E-2)	Cytokine activity (GO:0005125) (4.5E-3)	Colorectal cancer (2.3E-6)
6	Nef Tat env_gp120env_gp41	CCL5 IFNG IL1B IL10 IL2 IL2RA IL6 TNF	Leukocyte migration (GO:0050900) (2.3E-11)	Extracellular space (GO:0005615) (1.6E-7)	Cytokine activity(GO:0005125) (7.4E-11)	Cytokine-cytokinereceptor interaction (8.9E-10)
7	Nef Tat Vpr env_gp120env_gp41	IFNG IL10 IL2 IL6 TNF	Regulation of immunoglobulinproduction (GO:0002637) (1.1E-11)	Extracellular space (GO:0005615) (8.2E-6)	Cytokine activity (GO:0005125) (4.9E-8)	Allograft rejection (1.3E-6)
8	Tat env_gp120 env_gp160 env_gp41	IL1A IL1B IL2 IL6 LCK	Positive regulation of protein transport (GO:0051222) (4.6E-7)	Extracellular space (GO:0005615) (5.9E-4)	Cytokine activity (GO:0005125) (1.3E-5)	Graft-versus-host disease(1.7E-6)
9	Nef Tat env_gp120 matrix	CCL3 IFNG IL6 TNF	Positive regulation of protein aminoacid phosphorylation(GO:0001934) (1.8E-9)	Extracellular space (GO:0005615) (8.2E-6)	Cytokine activity (GO:0005125) (4.9E-8)	Allograft rejection (1.3E-6)
10	Nef Tat Vpr env_gp120 env_gp41 matrix	IFNG IL6 TNF	Regulation of chemokine biosynthetic process (GO:0045073) (4.9E-7)	Extracellular space (GO:0005615) (2.9E-3)	Cytokine activity (GO:0005125) (2.2E-4)	Graft-versus-host disease(5.7E-5)
11	Tat Vpr env_gp120 retropepsin	BCL2 CASP3 CYCS PARP1	B cell homeostasis (GO:0001782) (2.4E-3)	Protein phosphatase type 2A complex (GO:0000159) (4.7E-3)	not found	Amyotrophic lateralsclerosis (ALS) (3.2E-4)
12	Tat Vpr env_gp120 matrix	CCL3 IFNG IL6 TNF	Regulation of chemokine biosynthetic process (GO:0045073) (1.5E-6)	Extracellular space (GO:0005615) (1.5E-4)	Cytokine activity (GO:0005125) (3.3E-6)	Cytokine-cytokine receptor interaction (1.4E-4)
13	Nef env_gp120 env_gp160 env_gp41	CD4 IL1B IL2 IL6	Positive regulation of T cell activation (activation(GO:0050870) (1.7E-7)	Extracellular space (GO:0005615) (8.3E-3) activity (GO:0008083) (4.5E-4)	Graft-versus-hostdisease (1.7E-4)	
14	Nef Tat Vpr env_gp120 retropepsin	BCL2 CASP3 PARP1	B cell homeostasis (GO:0050870) (1.7E-7)	Nuclear envelope (GO:0005635) (3.2E-2)	Transcription factorbinding (GO:0008134)(7.7E-2)	Amyotrophic lateralsclerosis (ALS) (2.1E-2)
15	Nef Tat env_gp120 env_gp160 env_gp41	IL1B IL2 IL6	Positive regulation of immunoglobulinsecretion (GO:0051024)(3.7E-4)	Extracellular space (GO:0005615) (5.4E-2)	Not found	Not found
16	Nef Tat Vpr env_gp120 env_gp160 env_gp41	IL2 IL6	Positive regulation of immunoglobulin secretion secretion (GO:0051024) (3.7E-4)	Extracellular space (GO:0005615) (5.4E-2)	activity (GO:0008083)(1.2E-2)	Graft-versus-host disease (7.7E-3)
17	Nef Vpr Vpu env_gp120	CD4 CASP3 NFKB1	Regulation of T cellactivation (GO:0050863)(1.7E-2)	Intracellular organellelumen (GO:0070013)(1.9E-2)	Proteinhomodimerization activity(GO:0042803) (5.1E-2)	Epithelial cell signaling in Helicobacter pyloriinfection (2.7E-2)
18	Tat Vpr env_gp120 env_gp160 retropepsin	BCL2 CYCS	Positive regulation of catalytic activity activity (GO:0043085) (3.8E-2)	Protein phosphatase type 2A complex (GO:0000159)	Amyotrophic lateralsclerosis (ALS) (1.0E-2)	
19	Nef env_gp160 env_gp41 matrix	CALM1 IL6	Positive regulation of DNA binding (GO:0043388) (5.2E-3)	Not found	Not found	Not found

**Table 3:** **The significant GO-terms GO-id and KEGG pathways found in the bicliques extracted from** ***HV*** **_** ***negative* matrix, considering interaction types and direction of the interactions**

**Biclique**	**HIV protein**	**Human protein**	**GO term (bp)**	**GO term (cc)**	**GO term (mf)**	**KEGG pathway**
1	env_gp120 env_gp160 env_gp41	MAN1B1 MGAT2 MAN2C1 MAN2A1 MAN2A2 MANBA GBA3 MAN2B2 GAA MAN2B1 MAN1A1 MAN1A2 MAN1C1 GCS1 GANAB GANC GBA2	Mannose metabolic process (GO:0006013) (4.5E-11)	Golgi apparatus part (GO:0044431) (6.9E-6)	Mannosidase activity (GO:0015923) (8.7E-25)	N-Glycan biosynthesis (1.0E-11)
2	Rev capsid matrixnucleocapsid p1 p6	UBB UBC UBD	Long-term strengthening of neuromuscular junction (GO:0042062) (7.4E-4)	Cytosolic small ribosomal subunit (GO:0022627) (3.1E-3)	Structural constituent of ribosome (GO:0003735) (1.3E-2)	Not found
3	Rev Tat matrix p6	MAPK1 MAPK3 UBB UBC UBD	Cell cycle (GO:0007049)(7.2E-4)	Nucleoplasm (3.3E-4)	MAP kinase activity (GO:0004707) (3.2E-3)	Dorso-ventral axisformation (1.5E-2)
4	RT Vif env_gp120	IFNA1 IFNA16 IFNA2 IFNA7	Response to virus (GO:0009615) (5.1E-7)	Extracellular space (GO:0005615) (1.5E-4)	Interferon-alpha/beta receptor binding (GO:0005132) (2.3E-10)	Regulation of autophagy (3.0E-7)
5	Rev Vpu matrix retropepsin	CSNK2A1 CSNK2A2 CSNK2B	Wnt receptor signaling pathway (GO:0016055) (GO:0016055) (9.6E-5)	Not found	Protein serine/threoninekinase activity (Adherensjunction (2.3E-4)	
6	Rev Tat Vif matrix p6	MAPK1 MAPK3	Ras protein signaltransduction (GO:0007265) (7.8E-3)	Nucleolus (GO:0005730)(5.5E-2)	MAP kinase activity (GO:0004707) (1.1E-3)	Dorso-ventral axis formation (4.9E-3)
7	RT Rev Vpu matrix retropepsin	CSNK2A1 CSNK2B	Wnt receptor signaling pathway (GO:0016055) (9.8E-3)	Not found	Protein serine/threonine kinase activity (GO:0004674) (3.3E-2)	Adherens junction (1.5E-2)
8	RT Rev matrix	CSNK2A1 CSNK2B PRKCA	Wnt receptor signaling pathway (GO:0016055) (2.0E-2)	Not found	Protein serine/threonine kinase activity (GO:0004674) (1.1E-3)	Tight junction junction (6.9E-4)
9	Tat integrase matrix	KPNB1 RANBP5 TNPO1	Protein import into nucleus, docking (GO:0000059) (1.3E-3)	Nuclear pore (GO:0005643) (6.2E-3)	Nuclear localization sequence binding (GO:0008139) (5.4E-4)	Not found
10	Rev Tat matrix	MAPK1 MAPK3 PRKCA UBB UBC UBD	Regulation ofsynaptogenesis(GO:0051963) (1.7E-5)	Cytosol (GO:0005829)(1.2E-4)	MAP kinase activity (GO:0004707) (4.3E-3)	Aldosterone-regulated sodium reabsorption (6.3E-5)
11	Nef env_gp120 env_gp160	CD4 ITGAL ICAM1 HLA-DRB1 PRKCQ LCK	T cell activation (GO:0006468) (7.8E-6)	Plasma membrane part (GO:0044459) (1.5E-4)	Glycoprotein binding (GO:0001948) (1.4E-2)	Cell adhesion molecules (CAMs) (1.6E-4)
12	Tat capsid env_gp120	IFNG CD3D CD3E CD3G	T cell activation (GO:0042110) (2.6E-4)	Alpha-beta T cell recep- tor complex (GO:0042105) (2.2E-7)	T cell receptor binding (GO:0042608) (6.9E-4)	T cell receptor signaling pathway (9.3E-6)
13	Nef env_gp120 env_gp41	CXCR4 CD4	Initiation of viral infection (GO:0019059) (1.6E-3)	Not found	Coreceptor activity (GO:0015026) (1.5E-3)	Not found
14	Nef Tat env_gp120	TP53 ICAM1	T cell activation during immune response (GO:0002286) (9.6E-4)	Not found	Not found	Not found
15	Nef Tat Vpr	CDK9 TP53	Transcription,DNA-dependent(GO:0006351) (2.2E-2)	Nucleoplasm part (GO:0044451) (4.3E-2)	Not found	Not found
16	Nef RT Tat	PRKCA TP53	Induction of apoptosis by intracellular signals (GO:0008629) (4.0E-3)	Nsoluble fraction (GO:0005626) (6.6E-2)	Not found	Non-small cell lung cancer (1.1E-2)
17	Tat env_gp120 env_gp160	CD28 ICAM1	Regulation of immune effector process (GO:0002697) (7.5E-3)	External side of plasma membrane (GO:0009897) (1.3E-2)		Viral myocarditis (1.4E2)
18	Tat Vpr env_gp120	TP53 NFKB1	Regulation of specific transcription from RNA polymerase II promote(GO:0006357)(6.9E-3)	Nucleoplasm (GO:0005654) (3.3E-4)	Promoter binding (GO:0010843) (4.4E-3)	Pancreatic cancer (1.4E-2)
19	Tat env_gp120 env_gp41	CCL5 IFNG	Leukocyte chemotaxis (GO:0030595) (2.7E-3)	Extracellular space (GO:0005615) (5.4E-2)	Cytokine activity (GO:0005125) (1.5E-2)	Cytokine-cytokinereceptor interaction (5.2E-2)

**Figure 6:**
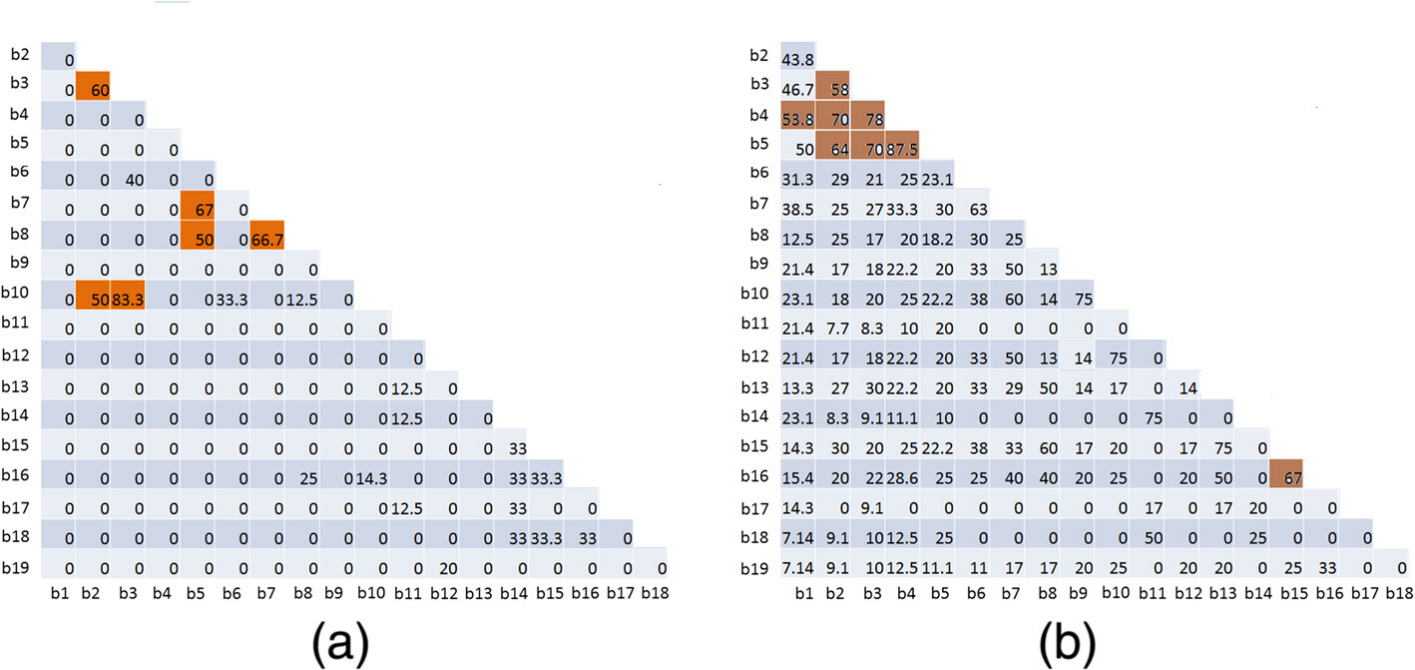
**Overlap score between all pairs of biclusters extracted from** ***HV*_*negative* (shown in (a)) and** ***HV*_*positive* (shown in (b)).**

A careful observation on Tables 2 and 3 reveals that some of the biclusters share some common proteins. We compute a overlap score between each pair of biclusters for detecting the amount of overlap between them. Overlap score between a pair of biclusters is defined as the number of common human proteins divided by the total number of unique human proteins in these biclusters. Figure 6(a) and (b) show the overlaps of the biclusters extracted from *HV*_ *positive* and *HV*_*negative* matrices respectively. From Figure 6(a) we can observe that the biclusters 5, 7 and 8 have substantial amount of overlaps among human proteins. From Table 3 it can also be noticed that the GO-terms associated with these biclusters are almost same. This is not quite unexpected because biclusters 5, 7 and 8 are enriched with casein kinase-2 protein family. It is established that HIV-1 transcription is regulated by casein kinase-2 protein family. Casein kinase-2 phosphorylates cellular proteins are involved in HIV-1 transactivation that contain multiple casein kinase-2 phosphorylation consensus sequences [19]. Similarly in Figure 6(b) we see that biclusters 3, 4 and 5 have multiple proteins common among them. From Table 2 it can be also found that GO-terms associated with these biclusters under biological process and cellular component are almost same.

From Figure 6 it can be observed that the biclusters from *HIV*_*positive* matrix show more overlaps than biclusters from *HIV*_*negative* matrix. But if we give a closer look on the number of proteins in each bicluster then it seems to be so obvious. In *HV*_ *positive* matrix, each bicluster contains 5.21 human proteins on average, whereas in *HV*_*negative* matrix, each bicluster contains only 3.78 human proteins on average. Moreover, the number of unique human proteins in all biclusters is surprisingly different in *HV*_ *positive* (approx 26%) and *HV*_*negative* (approx 70%) sets. Hence it is evident that overlaps among the *HV*_ *positive* biclusters are much greater than that among the *HV*_*negative* biclusters. From these observations we can conclude that the human proteins participating in the *HV*_*negative* biclusters are more diverse than that in the *HV*_ *positive* biclusters.

In Table 2 the first biclique consists of 14 human proteins BCL2, CASP3, TP53, IFNG, IFNG, IL10, IL2, IL6, MAPK1, NFKB1, PARP1, FOS, JUN and TNF that belong to the T cell receptor signaling pathway which plays a key role in human immune system. Latent HIV proviruses are thought to be primarily reactivated in vivo through stimulation of the T-cell receptor (TCR). Activation of the T-cell receptor (TCR) induces multiple signal transduction pathways, that leads to the ordered nuclear migration of the HIV transcription initiation factors NF-kB (nuclear factor kB) and NFAT (nuclear factor of activated T-cells) [20]. Human proteins in biclique 3 and 4 also belong to the same signaling pathway. Human proteins in biclique 5 are BCL2, CYCS, IFNG, IL2, IL6, MAPK1, FOS and JUN that are affected by two envelope glycoprotein GP120 and GP160, Transactivating regulatory protein (Tat) and accessory protein Vpr of HIV-1 virus that may lead to Colorectal cancer. The human proteins in biclique 6 interact with 4 HIV-1 proteins (2 envelop glycoprotein, Nef and Tat) and are involved in Cytokine-cytokine receptor interaction pathway. Cytokines are soluble extracellular proteins or glycoproteins that are crucial intercellular regulators and mobilizers of cells engaged in innate as well as adaptive inflammatory host defenses, cell growth, cell death, angiogenesis, and development and repair processes aimed at the restoration of homeostasis ( http://www.genome.jp/kegg/pathway/hsa/hsa04060.html). Human proteins in some bicliques are involved in Graft-versus-host disease in which a lethal complication of allogeneic hematopoietic stem cell transplantation (HSCT) is noticed where immunocompetent donor T cells attack the genetically disparate host cells. The importance of HIV-1 envelop glycoprotein in preventing the Graft-versus-host disease has recently been studied in [21]. The proteins in bicliques 11, 14, 18 are involved in the pathway Amyotrophic lateral sclerosis (ALS) which is caused by progressive, lethal, degenerative disorder of motor neurons. It is established that HIV causes diverse disorders of the brain, spinal cord and peripheral nerves. HIV infection could be a risk factor for either amyotrophic lateral sclerosis (ALS) itself or other motor neuron diseases [22].

In Table 3 biclique 1 consists of 3 HIV-1 envelop glycoproteins (env gp120, env gp160, and env gp41) and 17 human proteins which are associated with molecular function mannosidase activity and are also involved in the pathway N-Glycan biosynthesis. Recent studies have shows that the HIV-1 N-glycan composition plays a crucial role in the balance between dendritic cell (DC)-mediated antigen degradation and presentation and DC-mediated virus transmission to target cells [23]. The human proteins in biclique 4 are found to be involved in the regulation of autophagy. Autophagy is an intracellular lysosomal (vacuolar) degradation process that is characterized by the formation of double-membrane vesicles, known as autophagosomes, and it is involved in cell growth, survival, development and death. In [24] it is argued that HIV-1 infection can down-regulate autophagy in infected cells during acute infection. We find in bicliques 5 and 7, human proteins are belonging to the Adherens junction pathway which is the most common type of intercellular adhesions, and are important for maintaining tissue architecture and cell polarity and can limit cell movement and proliferation. Adherens junction consists oftransmembrane cadherins and cytoplasmic attached *α*-catenins and *β*-catenins assembled together into a multiprotein complex. This complex organization of cadherin-catenins and cytoskeleton strengthens cell-cell adhesion and has a role in signal transduction. Indirect evidence suggests that adherens junction may be involved in HIV-1 induced dysfunction of the vascular endothelium [25].

### Analysis of predicted rules

**Figure 7:**
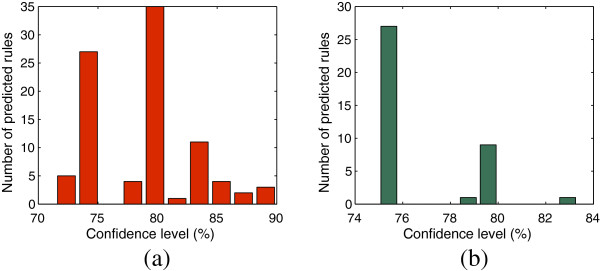
**Distribution of number of predicted rules extracted from** ***HV*_*positive* (shown in (a)) and** ***HV*_*negative* (shown in (b)) at different confidence level.**

We have predicted a total of 93 (62 rules are from the biclusters of *HV*_ *positive* and 31 rules are from the biclusters of *HV*_*negative* matrices) type-1 rules and 33 type-2 rules (among them 26 are from *HV*_ *positive* and 7 are from *HV*_*negative*). We studied the distribution of the confidence levels of these predicted rules. Figure 7 shows the distribution of the number of predicted rules at different confidence levels. From Figure 7(a) and (b) we can notice a significant change in the number of predicted rules when confidence level is changing from 80% onward for HV_ positive and the same situation is happening for HV_negative when confidence level changes from 75% onward. For extracting more biologically relevant rules we set the confidence level threshold at 80% for type-1 rules and 75% for type-2 rules. From Figure 7(a) we find that among 88 rules 58 rules have the confidence level above 79% whereas from Figure 7(b) we notice all the 38 rules have their confidence level above 75%. All the predicted type-1 and type-2 rules can be found in the Additional file 2. Here we show the type-1 rules predicted from *HV*_ *positive*, that have the confidence level 85% or above, and 7 type-2 rules predicted from *HV*_*negative* that have confidence level 75% or above in Tables 4 and 5 respectively. All the type-1 rules are important for getting valuable information about the regulation mechanism of human proteins. Those rules also say that regulation of some proteins triggered the regulation of other proteins with a high probability. So a proper analysis of these rules reveals the interdependence of the regulation mechanism of a set of proteins constituting a rule. For predicting the type-2 rules from the biclusters found in *HV*_ *positive* and *HV*_*negative* we treat human proteins as rows and viral proteins as columns in those biclusters. These type-2 rules are also important for explanation of the predicted interactions between some HIV-1 proteins and human proteins. So proper analysis of those type-1 and type-2 rules gives a wider aspect in regulation mechanism and prediction of interactions between HIV-1 and human proteins.

**Table 4:** Predicted rules generated from the biclusters (treating viral proteins as rows and human proteins as columns) found in HV_ positive matrix

**Sl no.**	**Association rules**	**Confidence**
Rule-1	[IFNG, IL6, TNF_UPREGULATES] ⇒ [IL10_UPREGULATES,IL2_DOWNREGULATES]	83.333
Rule-2	[IL6_UPREGULATES] ⇒ [IFNG,TNF_UPREGULATES]	85.715
Rule-3	[BCL2_DOWNREGULATES] ⇒ [CASP3_ACTIVATES, PARP1_INDUCES CLEAVAGE OF]	83.333
Rule-4	[CASP3_ACTIVATES] ⇒ [BCL2_DOWNREGULATES,PARP1_INDUCES CLEAVAGE OF]	83.333
Rule-5	[IL2_DOWNREGULATES] ⇒ [IL1B,IL6_UPREGULATES]	83.333
Rule-6	[IL2_DOWNREGULATES, IL6_UPREGULATES] ⇒ [IL1B_UPREGULATES]	83.333
Rule-7	[IL6_UPREGULATES] ⇒ [IL2_DOWNREGULATES]	85.714
Rule-8	[BCL2_DOWNREGULATES] ⇒ [CYCS_INDUCES RELEASE OF]	83.333
Rule-9	[IL6_UPREGULATES] ⇒ [IFNG,TNF_UPREGULATES]	85.714
Rule-10	[BCL2_DOWNREGULATES] ⇒ [CASP3_ACTIVATES,PARP1_INDUCES CLEAVAGE OF]	83.333
Rule-11	[CASP3_ACTIVATES] ⇒ [BCL2_DOWNREGULATES,PARP1_INDUCES CLEAVAGE OF]	83.333
Rule-12	[IL2_DOWNREGULATES] ⇒ [IL1B, IL6_UPREGULATES]	83.333
Rule-13	[IL2_DOWNREGULATES, IL6_UPREGULATES] ⇒ [IL1B_UPREGULATES]	83.333
Rule-14	[IL6_UPREGULATES] ⇒ [IL2_DOWNREGULATES]	85.714
Rule-15	[BCL2_DOWNREGULATES] ⇒ [CYCS_INDUCES RELEASE OF]	83.333

**Table 5:** Predicted rules generated from the biclusters (treating human proteins as rows and viral proteins as columns) found in HV_negative matrix

**Sl no.**	**Association rules**	**Confidence (%)**
Rule-1	[env_gp160,env_gp41] ⇒[env_gp120]	79.2
Rule-2	[matrix,nucleocapsid] ⇒[Rev,Tat,capsid,p1,p6]	75
Rule-3	[nucleocapsid,p6] ⇒[Rev,Tat,capsid,matrix,p1]	75
Rule-4	[Rev,Tat,matrix] ⇒[p6]	83.3
Rule-5	[capsid,env_gp120] ⇒[Tat]	80
Rule-6	[RT,env_gp120] ⇒[Vif]	80
Rule-7	[Vif,env_gp120] ⇒[RT]	80

### Predicted interactions

From the biclusters found from two matrices *HV*_ *positive* and *HV*_*negative* we predict some highly confident interactions between HIV-1 and human proteins. We also analyze the biological relevance of those interactions and conduct a literature survey to establish experimental evidence supporting our predicted interactions.

From the *HV*_ *positive* matrix 64 interactions between 8 HIV-1 and 31 human proteins and from *HV*_*negative* matrix 50 interactions between 13 HIV-1 proteins and 32 human proteins are predicted. For finding the experimental evidences of our predicted interactions we have extensively searched PUBMED for finding some recent reports describing the predicted interactions. The references of the articles we find from PUBMED showing the proof of our predictions are listed in Additional file 3. Among the 64 interactions 35 interactions and among 50 interactions 24 interactions are found to be experimentally validated and these are shown in Tables 6 and 7 with corresponding PUBMED ids.

**Table 6:** Predicted interactions found from biclusters constructed using rows as viral proteins and columns as human proteins (Sl. No. 1 to 26) and rows as human proteins and columns as viral proteins (Sl. No. 27 to 36)

**Sl. No.**	**HIV-1 Protein**	**Human protein**	**Interaction types**	**Pubmed Id**
1	Tat	CD4	DOWNREGULATES	22421574, 22342181
2	Tat	MAPK14	ACTIVATES	20378550
3	Tat	CASP9	ACTIVATES	11509621
4	Tat	CASP3	ACTIVATES	17505978
5	Tat	IL6	UPREGULATES	17151125, 9169458
6	Tat	CD4	INTERACTS WITH	12457987
7	Tat	PARP1	INDUCES CLEAVAGE OF	15498776
8	Tat	BCL2	DOWNREGULATES	11994280
9	Nef	JUN	ACTIVATES	12419805
10	Nef	FOS	ACTIVATES	20068037, 10388555
11	Nef	MAPK1	ACTIVATES	21738584
12	Nef	LCK	ACTIVATES	16849330
13	Nef	CASP3	ACTIVATES	11123279
14	Nef	IFNG	DOWNREGULATES	21858117
15	Nef	BCL2	DOWNREGULATES	15858021
16	Nef	CCL3	DOWNREGULATES	20015995
17	Nef	IL12B	UPREGULATES	19019824
18	Nef	IL6	UPREGULATES	11519483, 8799208
19	matrix	IL10	UPREGULATES	18178611
20	matrix	IL1B	UPREGULATES	18593760
21	matrix	IL2	DOWNREGULATES	21482826
22	env_gp120	CASP3	ACTIVATES	16330530
23	env_gp120	CD4	DOWNREGULATES	22226668
24	env_gp160	TNF	UPREGULATES	8938574
25	Vpu	BC+L2	DOWNREGULATES	11696595
26	env_gp120	MAPK8	ACTIVATES	11468147
27	env_gp120	TNF	INHIBITS	16873189
28	Tat	NFKBIA	UPREGULATES	22187158
29	Tat	IFNB1	UPREGULATES	9223731
30	Tat	CXCR4	INTERACTS WITH	11594685
31	Tat	CCL3	UPREGULATES	15204927
32	Nef	NFKB1	INTERACTS WITH	12419805
33	Nef	BCL2L1	DOWNREGULATES	11123279
34	Vpr	CASP9	ACTIVATES	12096338
35	Vpr	CYCS	INDUCES RELEASE OF	16511342

Direction of the regulations are taken from viral to human proteins.

**Table 7:** Predicted interactions found from biclusters constructed using rows as human proteins and columns as viral proteins (Sl. No. 1 to 10) and rows as viral proteins and columns as human proteins(Sl. No. 11 to 24)

**Sl. No.**	**HIV-protein**	**Human protein**	**Interaction types**	**Pubmed id**
1	env_gp160	CCL4	Inhibited by	21118814
2	env_gp160	CCL5	Inhibited by	21118814
3	env_gp160	HDAC6	Inhibited by	16148047
4	Rev	IPO7	Imported by	16704975
5	capsid	IPO7	Imported by	20147401
6	Rev	CD4	Inhibited by	8573391
7	matrix	NFKBIA	inhibited by	10722660
8	Capsid	APOBEC3G	Interacts with	17065315
9	Vif	TP53	Interacts with	21071676
10	RT	CD4	Interacts with	22426469
11	Vif	MAPK3	Phosphorylated by	10074203
12	Vif	UBB	Ubiquitinated by	15781449
13	Vif	UBD	Ubiquitinated by	18596088
14	Vif	MAPK1	Ubiquitinated by	10074203
15	Gag_Pr55	IFNA16	Inhibited by	11197304
16	Gag_Pr55	IFNA7	Inhibited by	8553538
17	Tat	CD4	Interacts with	12457987
18	Tat	PRKCQ	Interacts with	9446795
19	Tat	LCK	Interacts with	18854243
20	p6	MAPK3	Phosphorylated by	11773377
21	p6	MAPK1	Phosphorylated by	15155723
22	Gag_Pr55	IFNA2	Inhibited by	8553538
23	env_gp160	TP53	Interacts with	19023333
24	Nef	CD28	Interacts with	21819585

Here the direction of the interactions are taken from human to viral proteins.

The HIV-1 protein Trans-Activator of Transcription (TAT) contains a protein transduction domain, which allows Tat to enter cells by crossing the cell membrane causing infection and is therefore known as a cell penetrating peptide. Here we predict 14 human proteins that interact with Tat protein with specific interaction types. In row 1 of Table 6 we predict the downregulation of human CD4 cell by HIV-1 protein Tat. In [26] it is established that the downregulation of CD127 expression in HIV infection may be due to HIV protein Tat. In HIV infection, decreased CD127 expression on T-cells is correlated with reduced CD4(+) T-cell counts, increased viral replication and immune activation [26]. We also predict that Tat activates caspase-3 (CASP3) and caspase-9 (CASP9). In [27] it has been found that Tat activated both caspase-3 and endonuclease-G, a caspase-independent effector of apoptosis. We predict upregulation of human protein Interleukin 6 (IL6) by HIV-1 protein Tat and Nef. Tat induces the production of human interleukin-6 (huIL-6) and its receptor (huIL-6Ra) and activate STAT3 signaling [27]. In row 2 of Table 6 we can notice that activation of MAPK14 protein is mediated by Tat. In [28] it is also supported that Tat-mediated p66shc protein transduction augments TNF-a-induced p38 MAPK phosphorylation in endothelial cells. Row 7 of Table 6 indicates that Tat induces the cleavage of human protein Poly(ADP-ribose) polymerase 1 (PARP1). In [29] PARP1 is established as a negative regulator of HIV-1 transcription through competitive binding with Tat or the Tat.P-TEFb complex to TAR RNA (Trans-activation response element (TAR) RNA). The positive transcription elongation factor, P-TEFb, which plays an essential role in the regulation of transcription by RNA polymerase II (Pol II) is targeted by the Tat protein which bypasses normal cellular P-TEFb control and directly brings P-TEFb to the promoter proximal paused polymerase in the HIV genome and forms a complex Tat.P-TEFb. PARP-1 has a high affinity for TAR RNA and binds to the loop region of TAR RNA and displaces Tat or Tat.P-TEFb from the RNA [29]. In row 8 of Table 6 we have also predicted that Tat downregulates human protein BCL2 (B-cell lymphoma 2). In [30] it is noticed that Tat decreases the ratio of anti- and pro-apoptotic proteins, Bcl2/Bax. In [30] the author hypothesized that morphine enhances ‘HIV-Tat induced toxicity’ in human neurons and neuroblastoma cells. Enhanced toxicity by Tat and morphine was accompanied by increased numbers of TUNEL positive apoptotic neurons elevated caspase-3 levels and decreased ratio of anti- and pro-apoptotic proteins, Bcl2/Bax [30]. Nuclear factor (NF)-kB is a master regulator of pro-inflammatory genes and is upregulated by Tat as shown in row 29 of Table 6. HIV-1 Tat transactivator activates NF-kB by hijacking the inhibitor IkB-a and by preventing the repressor binding to the NF-kB complex [31]. CXCR-4 is an alpha-chemokine receptor specific for stromal-derived-factor-1 (SDF-1 also called CXCL12), a molecule endowed with potent chemotactic activity for lymphocytes. This receptor is one of several chemokine receptors that HIV isolates can use to infect CD4+ T cells. In row 30 of Table 6 we show that HIV-1 protein Tat interacts with CXCR-4. In [32] the HIV-1 Tat protein has been described as a ‘natural’ CXCR4 antagonist with anti-HIV-1 activity. Chemokine (C-C motif) ligand 3 (CCL3) is a protein which is encoded by the CCL3 gene. Chemokines are important mediators of inflammation. In Table 6 we predict that Tat upregulates CCL3. In [33] it has been demonstrated that the chemokine expression is dramatically increased in both the sera and brain of HIV-1 infected individual. The HIV-1 protein Tat has been detected in the central nervous system (CNS) of HIV infected individuals, and has induced chemokines from various cells within the brain. In [33] the authors speculated that the possible reason behind the dramatic increase in the secretion of the chemokines CCL2, CXCL8, CXCL10, CCL3, CCL4, and CCL5 is the interaction of human microglia, the resident phagocytes of the brain, with HIV-1 protein Tat.

Nef (Negative Regulatory Factor) is a HIV-1 protein which functions to manipulate the host’s cellular machinery and thus allow infection, survival and replication of the pathogen. Our prediction includes 12 human proteins that interact with Nef (5 proteins activated, 4 proteins downregulated, 2 proteins upregulated, and 1 interacted with).

We can notice in row 9 of Table 6 that Nef activates human protein JUN. In [34] a time- and dose-dependent increase in JNK activation accompanied with increased AP-1 activation, was observed by Nef protein. The c-Jun N-terminal kinases (JNKs) is originally a kinase protein that binds to c-JUN within its transcriptional activation domain. Other human proteins like FOS, MAPK1, LCK and CASP3 are predicted to be activated by Nef protein. In [35] Nef has been found to reduce the expression of anti-apoptotic proteins like BCL2, and activate the apoptotic hallmark like mitochondrial depolarization, activation of caspase-3, and cleavage of the caspase target poly(ADP-ribose) polymerase. These findings also support our prediction ‘Nef downregulates BCL2’. In rows 17 to 21 of Table 6 we predict 5 interactions between some common families of interleukin proteins with HIV-1 protein Nef and matrix. Interleukins are a group of cytokines and its large portions are responsible for the development of human immune system. Poor production of Th1-type cytokines including interleukin-12 (IL-12) is generally observed in CD4+T cells during the acute immunodeficiency syndrome associated with HIV-1 progression [36]. Cellular immunity is critically depended on Interleukins and its production is significantly decreased during HIV infection.

Our predictions also include other HIV-1 proteins like Vpr, matrix, Vpu, Envelop glycoprotein-120, and glycoprotein-160 that interact with some human proteins associated with specific interaction types. Our predicted interaction set also shows interactions between some common family of Caspases or cysteine-aspartic proteases which belong to family of cysteine proteases, with HIV-1 proteins Tat, Nef and Vpr. We notice that in our predicted interaction set Caspase proteins like CASP3 and CASP9 are activated by HIV-1 protein Tat, Nef and Vpr. The sequential activation of Caspase 3 has an impact in the execution phase of ‘Cell apoptosis’ which is commonly known as the process of ‘programmed cell death’. This suggests that Tat, Nef and Vpr are involved in many biological activities relating to the activation of Caspase family proteins which subsequently leads to apoptosis and programmed cell death.

In several studies it is established that Mitogen-activated protein kinase (MAPK) signal pathway is responsible for acting as a positive regulator of HIV-1 replication cycle. MAPK1, MAPK8 and MAPK14 which belong to the MAPK kinase family, are involved in different biological and cellular processes such as proliferation, differentiation, transcription regulation and development. From Table 6 we can notice that MAPK1, MAPK8 and MAPK14 are activated by HIV-1 proteins Nef, Tat and Env_Gp120 respectively. This suggests that HIV-1 proteins Nef, Tat and Env_gp120 have an increased effect in different biological and cellular processes that are responsible for the activation of MAPK kinase.

We are able to find PUBMED ids of some recent articles indexed in PUBMED that also agree with these predicted interactions. In Table 7, we show a total of 24 predicted interactions in which the direction of regulation of those interactions are from human proteins to viral proteins. The interactions in this direction are valuable as these types of interactions are useful for predicting human proteins which may prevent HIV infection. The predicted human proteins that are participating in these types of interactions are likely to be responsible for blocking HIV infection. Some recent reports whose PUBMED ids are listed in column 5 of Table 7, support this fact. For example, we predict that envelop glycoprotein 160 is inhibited by human proteins CCl4, CCl5 and HDAC6. In [37] the first two interactions are fully supported. The authors also investigated the mechanisms whereby nonpeptidic, low molecular weight CC chemokine receptor 5 (which is a G-protein-coupled receptor for the chemokines CCL3, CCL4, and CCL5) ligands block HIV-1 entry and infection. In [38] it is demonstrated that acetylation of alpha-tubulin is inhibited by the overexpression of active Histone deacetylase 6 (HDAC6). It is also established that Histone deacetylase 6 (HDAC6) prevents HIV-1 envelope-dependent cell fusion and infection without affecting the expression and codistribution of HIV-1 receptors [38]. As another example, we predict that HIV-1 virion infectivity factor (Vif) is phosphorylated by MAPK3. In [39] it is reported that the activation of mitogen-activated protein kinases (MAPK) through the Ras/Raf/MEK signaling pathway enhances the infectivity of HIV-1 virions infectivity factor (Vif). These evidences establish that many of our predicted interactions, which are not already included in HIV-1-human interaction database are supported by different literature. This demonstrates the utility of the proposed method.

**Figure 8:**
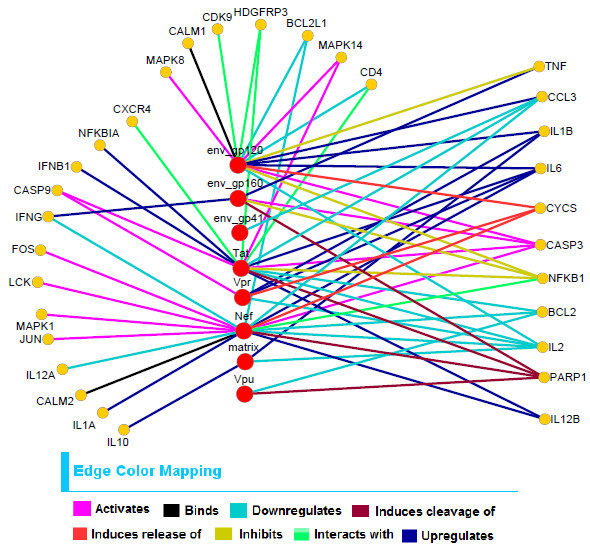
**The predicted bipartite network constructed from biclusters found in** ***HV*_*positive* matrix.** The big red circles denote viral proteins and small yellow circles denote human proteins that interact with these viral proteins. Here the edges are colored corresponding to the interaction types. These predicted interactions consist of 8 HIV-1 proteins and 31 human proteins.

**Figure 9:**
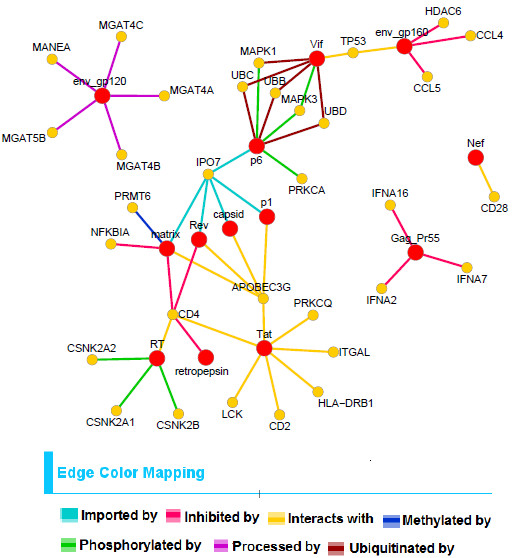
**The predicted bipartite network constructed from biclusters found in** ***HV*_*negative* matrix.** The big red circles denote viral proteins and small yellow circles denote human proteins that interact with these viral proteins. Here the edges are colored corresponding to the interaction types. These predicted interactions consist of 13 HIV-1 proteins and 32 human proteins.

In Figures 8 and 9, two bipartite networks that are constructed using our predicted interactions are shown. Figure 8 shows 64 predicted interactions found from the biclusters of *HV*_ *positive* matrix. Here 8 specific interaction types are shown in different colors. The big red nodes represent HIV-1 proteins and the yellow nodes represent the corresponding human proteins that are predicted to interact with these viral proteins by specific interaction types. Similarly Figure 9 shows 50 predicted interactions found from the biclusters of *HV*_*negative* matrix and 7 different interaction types are shown in 7 different colors.

### Detecting overlaps with other methods

**Figure 10:**
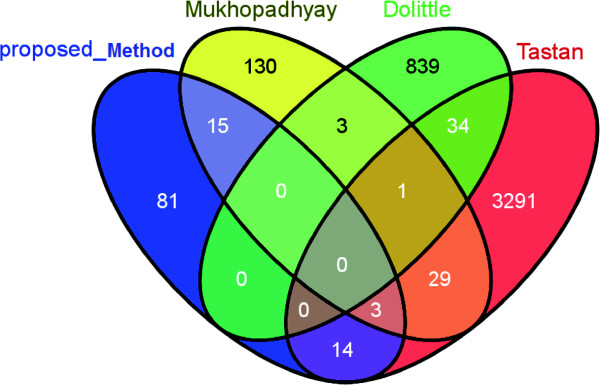
Venn diagram showing the overlap between the predicted interaction sets of four studies.

We have shown overlaps among the interactions predicted by Tastan et al. [9], Doolittle et al. [11], and Mukhopadhyay et al. [15] with our proposed method. This is shown in Figure 10. As these studies utilize extremely uncorrelated methodologies for prediction purpose, hence as expected, it is reflected on the overlap also. From this figure it appears that there is no reasonable overlaps between these three studies with our present study. Moreover we did not find reasonable overlaps among the other three studies also. Our present study has overlap of 18 and 17 interactions with that of Mukhopadhyay et al. [15] and Tastan et al. [9], respectively, but we do not find any interaction common with Dooloittle et al. [11]. Although Mukhopadhyay et al. [15] used association rule mining approach which is also utilized in the present study for prediction purpose, Venn diagram shows a little proportion of overlaps of interactions between Mukhopadhyay et al. and the present study. It is possibly due to the incorporation of interaction types and directionality in our present study. However the intuition behind detection of overlaps among several methods is not to consider these methods as competitive, but it could be more appropriate to consider them as collaborative in order to capture the full set of possible interactions and to put priority on the overlapped interactions. The predicted interactions which are supported by at least two studies are of great importance as these interactions are supported by more than one methodology. In the Additional file 4, we have listed all the interactions supported by two and three studies separately. Moreover, these methods have certain limitations for predicting the interactions so they are not expected to capture the same set of interactions.

**Figure 11:**
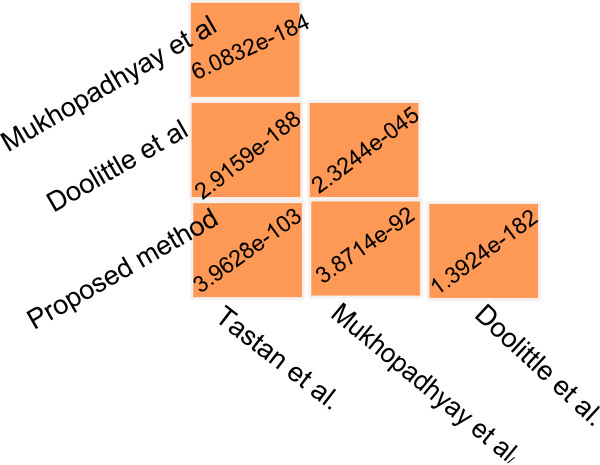
**This figure summarizes the results of Wilcoxon Ranksum test.** Here 4 random set of HIV-1–human protein pairs are generated. The size of 4 random sets is retained same as the size of predicted set of Tastan et al., Doolittle et al., Mukhopadhyay et al., and our present study. This is repeated for 500 times and overlaps between each pair of sets is calculated. The p-values shown in figure signify the result of Wilcoxon ranksum test between this random overlaps and the real overlaps.

However, we have performed a significance test to investigate whether the overlaps among all the studies is more than expected by random chance. As we are not aware of the distribution of overlaps, so a nonparametric test is the best option here. We have utilized Wilcoxon Ranksum test for this purpose. We have first created HIV-1–human protein pairs by randomly selecting HIV-1 proteins and human proteins from HHPID dataset. We have selected four sets of random pairs by retaining the size of each set same as the size of four predicted sets which are being tested. Next, we have computed the overlaps among each pair of random interaction sets. We performed this procedure 500 times and got 500 random overlaps for each pair of random sets. These are then compared with the real overlaps using Wilcoxon Ranksum test. The resulting p-values are shown in Figure 11. From this figure it is evident that the resulting p-values are significantly low in all cases of overlaps. This is strong evidence against the null hypothesis suggesting that the overlaps are significant. Hence it is evident that although the overlaps are small, still they are more than expected by random chance.

## Conclusions

Here we have posed the problem of identifying new regulatory interactions between HIV-1 and human proteins based on the existing PPI database as an association rule mining problem based on BiMax biclustering algorithm. For predicting new interactions here we consider the direction of regulation as well as the types of the interactions as reported in the HIV-1-human interaction database. Therefore, our predicted interaction set has some additional information along with the predicted pairs. It keeps record about the regulation direction and interaction type of all predicted pairs. It may substancially reduce the effort of molecular biologist as it does not require to explore all possible combinations of interaction types that could be possible for a predicted pair.

We have shown the overlaps among the predicted sets of interactions of present study with some other studies. All other studies have utilized completely different methodologies and possible prediction set of each study is utterly dependent on these methodologies. So, it is somewhat not justified to compare all these techniques together by considering only the predicted interaction sets produced by all these methods. For example Doolittle et al., exploited structural similarity information of HIV-1 and human proteins for prediction purpose. So, the human protein which does not show structural similarity with any possible HIV-1 proteins, can not be included in the possible prediction set. Moreover they have not used HHPID dataset for prediction, instead they utilized HPRD, PIG databases for collecting information about interactions, Dali and PDB databases for acquiring information about structural similarity and HHPID dataset for validating the predictions. Although Tastan et al., Mukhopadhyay et al., and our present study use HHPID dataset for prediction purpose but the main drawback of Mukhopadhyay et al. technique is that it cannot predict any interaction whose protein pairs are not included in a maximal biclique. Our present study also has the same limitation but with a little improvement that it keeps the interaction type and directionality information with each biclique. However Tastan et al., produces all possible pairs of interactions and are able to compute prediction score of each of the possible interaction pairs. But they are not able to provide the interaction type and directionality information associated with the predicted set of interactions.

For validating the predicted interactions some evidences from recent literature are collected to establish the fact that our predicted interactions are supported in different literature. We also performed a gene ontology based study on the predicted bicliques and found some significant pathways in which the human proteins of those bicliques are involved. Considering the regulation direction we have predicted two types of association rules at certain confidence levels and illustrated the general meaning of those types of rules. Here we have also predicted some human proteins that are immuned to certain HIV-1 attack. Type-2 rules are also equally important for prediction of new interactions between HIV-1 and human proteins.

Here we have not considered the PPI information among the host proteins for predicting PPIs between human and HIV-1 proteins. Biclustering in HIV-1–human PPI network yields strong interaction modules or bicliques between human and HIV-1 proteins. Association rules are extracted from these bicliques and predicted interactions are based on these predicted rules. So, for prediction purpose we only utilize viral-host interactions. It may be possible to integrate host PPI information along with the viral-host PPIs. The interactions between human proteins that form bicliques with viral proteins may be taken into consideration. This may contribute greater knowledge about the predicted interactions.

Also Similar type of analysis may be done on other type of host-pathogen networks. Host pathogen interaction networks that have sufficient information about the interaction type between host proteins and pathogen proteins can be similarly analyzed for host-pathogen interaction prediction.

In analyzing the type-1 rules we overlooked the effect caused by the downregulation or upregulation or activation of the proteins constituting the antecedent part of these rules. Some of these proteins may be act as a transcription factor to activate or repress the regulation of other human proteins and so on. Chaining through these regulatory pathways if we can find some human proteins that affect some viral proteins then we will be able to find a closed path which starts with some set of viral proteins and ends up with the same or different viral proteins through the regulation mechanism of proteins constituting this path. Analysis of these regulatory pathways may greatly contribute to our understanding of the process of HIV-1 replications and different stages of virus life cycle in human body. For this type of analysis we have to consider the whole regulatory network of human proteome besides the viral-host bipartite network. We suggest this as a future work plan for this work.

## Availability

The Additional file 1 and other related materials are available at http://kucse.in/hiv/.

## Competing interests

The authors declare that they have no competing interests.

## Authors’ contributions

AM did the initial planning and collected the dataset. AM and SR performed the data processing, developed the code, performed the analysis, and drafted the manuscript. UM provided constructive discussion, corrected the manuscript, and supervised the complete work. All the authors read and approved the final manuscript.

## Supplementary Material

Additional file 1 **Association rule mining based on biclustering.** Association rule mining that utilizes the biclustering technique is breifly described here. Click here for file [ http://kucse.in/hiv/supplementary_bioinfo1/association_rules_report.pdf]. Click here for file

Additional file 2 **All type-1 and type-2 rules.** All the predicted type-1 and type-2 rules are listed here. Click here for file

Additional file 3 **References of PUBMED entry.** The references of the articles we find from PUBMED showing the proof of our predictions are listed here. Click here for file http://kucse.in/hiv/supplementary_bioinfo1/reference_of_pubmed.pdf. Click here for file

Additional file 4 Predicted interactions supported by more than one methodology. Click here for file
