# Supplementary material for: Incorporating the type and direction information in predicting novel regulatory interactions between HIV-1 and human proteins using a biclustering approach
Source: BMC Bioinformatics. 2014 Jan 24;15:26. doi: 10.1186/1471-2105-15-26 (PMC3922888; doi:10.1186/1471-2105-15-26)
Supplement: Additional file 1 — Association rule mining based on biclustering. Association rule mining that utilizes the biclustering technique is breifly described here. Click here for file [ http://kucse.in/hiv/supplementary_bioinfo1/association_rules_report.pdf]. [file 1471-2105-15-26-S1.pdf]

1 ASSOCIATION RULE MINING

In data mining, association rule mining is a popular and well researched method for discovering interesting relations between variables and showing attribute-value associations that occur frequently in large databases (Bandyopadhyay *et al.*, 2005). The problem of association rule mining is defined as follows:

Let $I = \{i_1, i_2, \ldots i_n\}$ be a set of n items and X be an itemset where $X \subset I$. A k-itemset is a set of k items. Let $T = \{(t_1, X_1), (t_2, X_2), \ldots (t_m, X_m)\}$ be a set of m transactions, where t_i and X_i, $i = 1, 2, \ldots, m$ are the transaction identifier and the associated itemset respectively. The cover of an itemset X in T is defined as follows:

$$cover(X, T) = \{t_i | (t_i, X_i) \epsilon T, X \subset X_i\}$$

. The support of an itemset X in T is :

$$support(X, T) = |cover(X, T)|$$

and the frequency of an itemset is :

$$frequency(X, T) = \frac{support(X, T)}{|T|}$$

. The support of an itemset X is the number of transactions where all the items in X appear in each transaction. The frequency of an itemset is the probability of its occurrence in a transaction in T. An itemset is called frequent if its support in T is greater than some threshold *min_sup*. The collection of frequent itemsets with respect to a minimum support *min_sup* in T, denoted by $F(T, min\_sup)$ is defined as

$$F(T, minsup) = \{X \subset I, support(X, T) > min\_sup\}$$

. The objective of ARM is to find all rules of the form $X \Rightarrow Y, X \bigcap Y = \phi$ with probability c%, indicating that if itemset X occurs in a transaction, the itemset Y also occurs with probability c%. X and Y are called the antecedent and consequent of the rule respectively. Support of a rule denotes the percentage of transactions in T that contains both X and Y. This is taken to be the probability $P(X \bigcup Y)$. An association rule (AR) is called frequent if its support exceeds a minimum value *min_sup* and called confident if its confidence value exceeds a threshold *min_conf*. The confidence of a rule X ⇒ Y in T denotes the percentage of the transactions in T containing X that also contains Y. It is taken to be the conditional probability $P(Y|X)$. In other words,

$$confidence(X \Rightarrow Y, T) = \frac{support(X \bigcup Y, T)}{support(X, T)}$$

. A rule is called confident if its confidence value exceeds a threshold *min_conf*. Formally the ARM problem can be defined as: find the set of all rules R of the form $X \Rightarrow Y$ such that

$$R = \{X \Rightarrow Y | X, Y \subset I, X \bigcap Y = \phi, X \bigcup Y = F(T, min\_sup), confidence(X \Rightarrow Y, T) > min\_conf\}.$$

For generating the ARM the two general steps are: find all frequent itemsets and then generate strong ARs from the frequent itemsets Hipp *et al.* (2000). But the number of itemsets grows exponentially with the number of items $|I|$. A commonly used algorithm for generating frequent itemsets is the *Apriori* algorithm Agrawal and Srikant (1994). The computational complexity of *Apriori* becomes intractable for very low value of min_sup due to generation of large number of frequent itemset, when the number of items is very large. In this context the concept of frequent closed itemset Pasquier *et al.* (1999); Zaki and Hsiao (2005) is defined to improve the efficiency of mining rules ignoring the redundant information present in the frequent itemsets. The main property behind the usage of this concept in mining association rules is that the frequent closed itemset with supports constitute a non-redundant minimal representation of the frequent itemsets and their supports.

2 ASSOCIATION RULE MINING BASED ON BICLUSTERING

Biclustering technique is generally used in microarray gene expression data for identifying co-regulated genes under a particular set of experimental conditions (Mukhopadhyay *et al.*, 2010).

A gene expression data matrix A = (R,C) can be represented by a set of rows R and a set of columns C denoting genes and samples, respectively. Each entry in this matrix is denoted by (r, c) and the expression level of gene r in sample c is represented by $a_r c$, $r \epsilon R$, $c \epsilon C$. A bicluster can be defined as a submatrix B = (I, J) of matrix A (R,C) where $I \epsilon R$ and $J \epsilon C$, and subset of genes in the bicluster are similarly expressed over the subset of conditions and vice versa. The goal of the biclustering is to find submatrices, that is, subgroups of genes and subgroups of conditions, where the genes exhibit highly correlated activities for every condition. There are different kinds of biclusters available, viz., constant, row-constant, column-constant, additive pattern, multiplicative pattern, and combination of both additive and multiplicative patterns.

Besides the gene expression data matrix biclustering approach can also be applied in any data matrix to extract similar type of submatrices. For example biclusters in binary matrices represent submatrices that contain all 0 or all 1 values. Biclustering in binary matrices can be intelligently used in association rule mining technique. For example any transaction dataset with m transactions and n items can be represented as a binary matrix with rows representing transactions and columns representing items. An entry $t_{i,j}$ is '1' when the $j^t h$ item is purchased in the $i^t h$ transaction and '0' otherwise. In this matrix the columns of all-1 bicluster with *min_sup* number of rows represent a frequent itemset. For example consider a bicluster consisting of 5 human proteins (H_1, H_2,. . . , H_5) and 3 HIV-1 proteins (V_1,V_2 and V_3) and minimum support value is 3. Now the columns of these bicluster represent a frequent item-set with respect to this minimum support value. This situation is shown in figure . Now there are a total of 30 possible rules can be generated from this itemset by placing each combination of human proteins as antecedents or consequences. For illustrating the support and the confidence of those rules let us consider the rule-25. Now without loss of generality we may assume the proteins in the antecedent of this rule form another biclique (or bicluster) with 4 viral proteins: $V1$, $V2$, $V3$, and $V4$. So the confidence of this rule is 3/4 or 75%. Similarly we can compute the confidence of all the generated rules.

Among the various biclustering technique we use Binary inclusion-Maximal (BiMax) biclustering algorithm (Prelic, 2006) for identifying all maximal biclusters in the input binary matrix. As BiMax identifies all maximal bicluster (a maximal bicluster

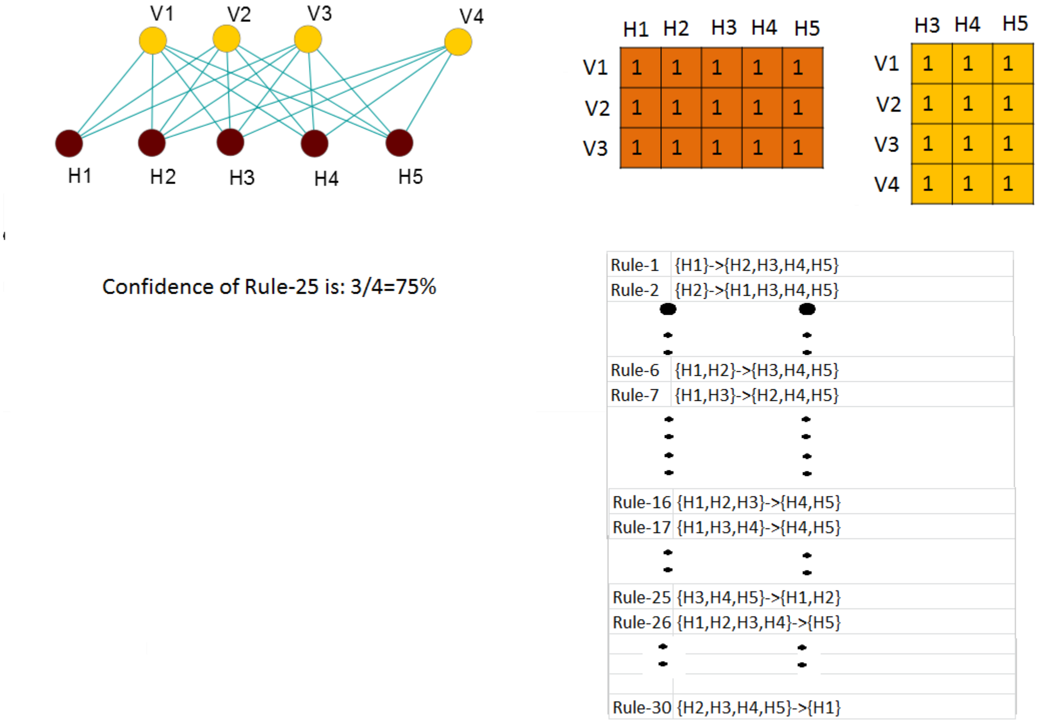

Fig. 1. Illustration of association rule mining based on biclustering technique

means that this bicluster is not a proper subset of any bicluster), and the columns of a maximal bicluster constitute the frequent closed itemset, so finding the all-1 biclusters that have at least *min_sup* number of rows are equivalent to find the set of frequent closed itemsets.

REFERENCES

Agrawal, R. and Srikant, R. (1994). Fast algorithms for mining association rules in large databases. In *Proc. 20th International Conference on Very Large Data Bases. San Francisco, CA, USA*, pages 487–499. Morgan Kaufmann Publishers Inc.

Bandyopadhyay, S., Maulik, U., Holder, L., and Cook, D. (2005). *Advanced Methods for Knowledge Discovery from Complex Data (Advanced Information and Knowledge Processing)*. Springer-Verlag, London.

Hipp, J., Guntzer, U., and Nakhaeizadeh, G. (2000). Algorithms for association rule mining a general survey and comparison. *SIGKDD Explorations*, **2**(58-64).

Mukhopadhyay, A., Maulik, U., and Bandyopadhyay, S. (2010). On biclustering of gene expression data. *Current Bioinformatics*, **5**, 204–216.

Pasquier, N., Bastide, Y., Taouil, R., and Lakhal, L. (1999). Discovering frequent closed itemsets for association rules. In *In: Proc. 7th International Conference on Database Theory (ICDT-99).*, pages 398–416.

Prelic (2006). A systematic comparison and evaluation of biclustering methods for gene expression data. *Bioinformatics*, **22**, 1122–1129.

Zaki, M. and Hsiao, C. (2005). Efficient algorithms for mining closed itemsets and their lattice structure. *IEEE Transactions on Knowledge and Data Engineering*, **17**, 462–478.
