# Supplementary material for: Incorporating the type and direction information in predicting novel regulatory interactions between HIV-1 and human proteins using a biclustering approach
Source: BMC Bioinformatics. 2014 Jan 24;15:26. doi: 10.1186/1471-2105-15-26 (PMC3922888; doi:10.1186/1471-2105-15-26)
Supplement: Additional file 3 — References of PUBMED entry. The references of the articles we find from PUBMED showing the proof of our predictions are listed here. Click here for file http://kucse.in/hiv/supplementary_bioinfo1/reference_of_pubmed.pdf. [file 1471-2105-15-26-S3.pdf]

1 REFERENCES OF THE PUBMED ID USED IN MAIN TEXT

REFERENCES

Agy, M. B., Acker, R. L., Sherbert, C. H., and Katze, M. G. (1995). Interferon treatment inhibits virus replication in HIV-1- and SIV-infected CD4+ T-cell lines by distinct mechanisms: evidence for decreased stability and aberrant processing of HIV-1 proteins. *Virology*, **214**(2), 379–386.

Akari, H., Bour, S., Kao, S., Adachi, A., and Strebel, K. (2001). The human immunodeficiency virus type 1 accessory protein Vpu induces apoptosis by suppressing the nuclear factor kappaB-dependent expression of antiapoptotic factors. *J. Exp. Med.*, **194**(9), 1299–1311.

Ambrosino, C., Ruocco, M. R., Chen, X., Mallardo, M., Baudi, F., Trematerra, S., Quinto, I., Venuta, S., and Scala, G. (1997). HIV-1 Tat induces the expression of the interleukin-6 (IL6) gene by binding to the IL6 leader RNA and by interacting with CAAT enhancer-binding protein beta (NF-IL6) transcription factors. *J. Biol. Chem.*, **272**(23), 14883–14892.

An, T., Ouyang, W., Pan, W., Guo, D., Li, J., Li, L., Chen, G., Yang, J., Wu, S., and Tien, P. (2012). Amino acid derivatives of the (-) enantiomer of gossypol are effective fusion inhibitors of human immunodeficiency virus type 1. *Antiviral Res.*

Arnold, M., Nath, A., Hauber, J., and Kehlenbach, R. H. (2006). Multiple importins function as nuclear transport receptors for the Rev protein of human immunodeficiency virus type 1. *J. Biol. Chem.*, **281**(30), 20883–20890.

Atasheva, S., Fish, A., Fornerod, M., and Frolova, E. I. (2010). Venezuelan equine Encephalitis virus capsid protein forms a tetrameric complex with CRM1 and importin alpha/beta that obstructs nuclear pore complex function. *J. Virol.*, **84**(9), 4158–4171.

Biggs, T. E., Cooke, S. J., Barton, C. H., Harris, M. P., Saksela, K., and Mann, D. A. (1999). Induction of activator protein 1 (AP-1) in macrophages by human immunodeficiency virus type-1 NEF is a cell-type-specific response that requires both hck and MAPK signaling events. *J. Mol. Biol.*, **290**(1), 21–35.

Borgatti, P., Zauli, G., Cantley, L. C., and Capitani, S. (1998). Extracellular HIV-1 Tat protein induces a rapid and selective activation of protein kinase C (PKC)-alpha, and -epsilon and -zeta isoforms in PC12 cells. *Biochem. Biophys. Res. Commun.*, **242**(2), 332–337.

Buriani, A., Petrelli, L., Facci, L., Romano, P. G., Dal Tosso, R., Leon, A., and Skaper, S. D. (1999). Human immunodeficiency virus type 1 envelope glycoprotein gp120 induces tumor necrosis factor-alpha in astrocytes. *J NeuroAIDS*, **2**(2), 1–13.

Crawley, A. M. and Angel, J. B. (2012). The influence of HIV on CD127 expression and its potential implications for IL-7 therapy. *Semin Immunol*.

D'Aversa, T. G., Yu, K. O., and Berman, J. W. (2004). Expression of chemokines by human fetal microglia after treatment with the human immunodeficiency virus type 1 protein Tat. *J. Neurovirol.*, **10**(2), 86–97.

De Clercq, E. and Schols, D. (2001). Inhibition of HIV infection by CXCR4 and CCR5 chemokine receptor antagonists. *Antivir. Chem. Chemother.*, **12 Suppl 1**, 19–31.

De Francesco, M. A., Baronio, M., and Poiesi, C. (2011). HIV-1 p17 matrix protein interacts with heparan sulfate side chain of CD44v3, syndecan-2, and syndecan-4 proteoglycans expressed on human activated CD4+ T cells affecting tumor necrosis factor alpha and interleukin 2 production. *J. Biol. Chem.*, **286**(22), 19541–19548.

DeHart, J. L., Bosque, A., Harris, R. S., and Planelles, V. (2008). Human immunodeficiency virus type 1 Vif induces cell cycle delay via recruitment of the same E3 ubiquitin ligase complex that targets APOBEC3 proteins for degradation. *J. Virol.*, **82**(18), 9265–9272.

Deregibus, M. C., Cantaluppi, V., Doublier, S., Brizzi, M. F., Deambrosis, I., Albini, A., and Camussi, G. (2002). HIV-1-Tat protein activates phosphatidylinositol 3-kinase/ AKT-dependent survival pathways in Kaposi's sarcoma cells. *J. Biol. Chem.*, **277**(28), 25195–25202.

Di Sabatino, A., Pickard, K. M., Rampton, D., Kruidenier, L., Rovedatti, L., Leakey, N. A., Corazza, G. R., Monteleone, G., and MacDonald, T. T. (2008). Blockade of transforming growth factor beta upregulates T-box transcription factor T-bet, and increases T helper cell type 1 cytokine and matrix metalloproteinase-3 production in the human gut mucosa. *Gut*, **57**(5), 605–612.

Ehret, A., Li-Weber, M., Frank, R., and Krammer, P. H. (2001). The effect of HIV-1 regulatory proteins on cellular genes: derepression of the IL-2 promoter by Tat. *Eur. J. Immunol.*, **31**(6), 1790–1799.

Fan, Z., Tardif, G., Hum, D., Duval, N., Pelletier, J. P., and Martel-Pelletier, J. (2009). Hsp90beta and p130(cas): novel regulatory factors of MMP-13 expression in human osteoarthritic chondrocytes. *Ann. Rheum. Dis.*, **68**(6), 976–982.

Fiala, M., Rhodes, R. H., Shapshak, P., Nagano, I., Martinez-Maza, O., Diagne, A., Baldwin, G., and Graves, M. (1996). Regulation of HIV-1 infection in astrocytes: expression of Nef, TNF-alpha and IL-6 is enhanced in coculture of astrocytes with macrophages. *J. Neurovirol.*, **2**(3), 158–166.

Fiume, G., Vecchio, E., De Laurentiis, A., Trimboli, F., Palmieri, C., Pisano, A., Falcone, C., Pontoriero, M., Rossi, A., Scialdone, A., Fasanella Masci, F., Scala, G., and Quinto, I. (2012). Human immunodeficiency virus-1 Tat activates NF-B via physical interaction with IB- and p65. *Nucleic Acids Res.*, **40**(8), 3548–3562.

Flory, E., Kunz, M., Scheller, C., Jassoy, C., Stauber, R., Rapp, U. R., and Ludwig, S. (2000). Influenza virus-induced NF-kappaB-dependent gene expression is mediated by overexpression of viral proteins and involves oxidative radicals and activation of IkappaB kinase. *J. Biol. Chem.*, **275**(12), 8307–8314.

Garcia-Perez, J., Rueda, P., Staropoli, I., Kellenberger, E., Alcami, J., Arenzana-Seisdedos, F., and Lagane, B. (2011). New insights into the mechanisms whereby low molecular weight CCR5 ligands inhibit HIV-1 infection. *J. Biol. Chem.*, **286**(7), 4978–4990.

Garg, H. and Blumenthal, R. (2006). HIV gp41-induced apoptosis is mediated by caspase-3-dependent mitochondrial depolarization, which is inhibited by HIV protease inhibitor nelfinavir. *J. Leukoc. Biol.*, **79**(2), 351–362.

Gibellini, D., Re, M. C., La Placa, M., and Zauli, G. (2002). Differentially expressed genes in HIV-1 tat-expressing CD4(+) T-cell line. *Virus Res.*, **90**(1-2), 337–345.

Hemonnot, B., Cartier, C., Gay, B., Rebuffat, S., Bardy, M., Devaux, C., Boyer, V., and Briant, L. (2004). The host cell MAP kinase ERK-2 regulates viral assembly and release by phosphorylating the p6gag protein of HIV-1. *J. Biol. Chem.*, **279**(31), 32426–32434.

Kamada, K., Igarashi, T., Martin, M. A., Khamsri, B., Hatcho, K., Yamashita, T., Fujita, M., Uchiyama, T., and Adachi, A. (2006). Generation of HIV-1 derivatives that productively infect macaque monkey lymphoid cells. *Proc. Natl. Acad. Sci. U.S.A.*, **103**(45), 16959–16964.

Kobayashi, M., Takaori-Kondo, A., Miyauchi, Y., Iwai, K., and Uchiyama, T. (2005). Ubiquitination of APOBEC3G by an HIV-1 Vif-Cullin5-Elongin B-Elongin C complex is essential for Vif function. *J. Biol. Chem.*, **280**(19), 18573–18578.

Kuzmina, A., Hadad, U., Fujinaga, K., and Taube, R. (2012). Functional characterization of a human cyclin T1 mutant reveals a different binding surface for Tat and HEXIM1. *Virology*, **426**(2), 152–161.

Leroux-Roels, I., Koutsoukos, M., Clement, F., Steyaert, S., Janssens, M., Bourguignon, P., Cohen, K., Altfeld, M., Vandepapeliere, P., Pedneault, L., McNally, L., Leroux-Roels, G., and Voss, G. (2010). Strong and persistent CD4+ T-cell response in healthy adults immunized with a candidate HIV-1 vaccine containing gp120, Nef and Tat antigens formulated in three Adjuvant Systems. *Vaccine*, **28**(43), 7016–7024.

Ma, W., Mishra, S., Gajanayaka, N., Angel, J. B., and Kumar, A. (2009). HIV-1 Nef inhibits lipopolysaccharide-induced IL-12p40 expression by inhibiting JNK-activated NFkappaB in human monocytic cells. *J. Biol. Chem.*, **284**(12), 7578–7587.

Messmer, D., Jacque, J. M., Santisteban, C., Bristow, C., Han, S. Y., Villamide-Herrera, L., Mehlhop, E., Marx, P. A., Steinman, R. M., Gettie, A., and Pope, M. (2002). Endogenously expressed nef uncouples cytokine and chemokine production from membrane phenotypic maturation in dendritic cells. *J. Immunol.*, **169**(8), 4172–4182.

Misse, D., Esteve, P. O., Renneboog, B., Vidal, M., Cerutti, M., St Pierre, Y., Yssel, H., Parmentier, M., and Veas, F. (2001). HIV-1 glycoprotein 120 induces the MMP-9 cytopathogenic factor production that is abolished by inhibition of the p38 mitogen-activated protein kinase signaling pathway. *Blood*, **98**(3), 541–547.

Miterski, B., Jaeckel, S., Epplen, J. T., Pohlau, D., and Hardt, C. (1999). The interferon gene cluster: a candidate region for MS predisposition? Multiple Sclerosis Study Group. *Genes Immun.*, **1**(1), 37–44.

Moon, H. S. and Yang, J. S. (2006). Role of HIV Vpr as a regulator of apoptosis and an effector on bystander cells. *Mol. Cells*, **21**(1), 7–20.

Muller, B., Patschinsky, T., and Krausslich, H. G. (2002). The late-domain-containing protein p6 is the predominant phosphoprotein of human immunodeficiency virus type 1 particles. *J. Virol.*, **76**(3), 1015–1024.

Muthumani, K., Zhang, D., Hwang, D. S., Kudchodkar, S., Dayes, N. S., Desai, B. M., Malik, A. S., Yang, J. S., Chattergoon, M. A., Maguire, H. C., and Weiner, D. B. (2002). Adenovirus encoding HIV-1 Vpr activates caspase 9 and induces apoptotic cell death in both p53 positive and negative human tumor cell lines. *Oncogene*, **21**(30), 4613–4625.

Nobile, C., Rudnicka, D., Hasan, M., Aulner, N., Porrot, F., Machu, C., Renaud, O., Prevost, M. C., Hivroz, C., Schwartz, O., and Sol-Foulon, N. (2010). HIV-1 Nef inhibits ruffles, induces filopodia, and modulates migration of infected lymphocytes. *J. Virol.*, **84**(5), 2282–2293.

Olivieri, K. C., Mukerji, J., and Gabuzda, D. (2011). Nef-mediated enhancement of cellular activation and human immunodeficiency virus type 1 replication in primary T cells is dependent on association with p21-activated kinase 2. *Retrovirology*, **8**, 64.

Table 1. Predicted Regulatory Interactions with their supporting PUBMED id and references.

SL.No.	Predicted Regulatory Interactions	PUBMED ID	Reference
1	Tat DOWNREGULATES CD4	22421574, 22342181	(Crawley and Angel, 2012; Kuzmina *et al.*, 2012)
2	Tat DOWNREGULATES IL2	20728522, 11385624	(Leroux-Roels *et al.*, 2010; Ehret *et al.*, 2001)
3	Tat ACTIVATES MAPK14	20378550	(Wong *et al.*, 2010)
4	Tat ACTIVATES CASP9	11509621	(Park *et al.*, 2001)
5	Tat ACTIVATES CASP3	17505978	(Zhao *et al.*, 2007)
6	Tat UPREGULATES IL6	17151125, 9169458	(Zeng *et al.*, 2007; Ambrosino *et al.*, 1997)
7	Tat INTERACTS WITH CD4	12457987	(Gibellini *et al.*, 2002)
8	Tat INDUCES CLEAVAGE OF PARP1	15498776	(Parent *et al.*, 2005)
9	Tat DOWNREGULATES BCL2	11994280	(Deregibus *et al.*, 2002)
10	Nef ACTIVATES JUN	12419805	(Varin *et al.*, 2003)
11	Nef ACTIVATES FOS	20068037, 10388555	(Biggs *et al.*, 1999)
12	Nef ACTIVATES MAPK1	21738584	(Sarmady *et al.*, 2011)
13	Nef ACTIVATES LCK	16849330	(Trible *et al.*, 2006)
14	Nef ACTIVATES CASP3	11123279	(Rasola *et al.*, 2001)
15	Nef DOWNREGULATES IFNG	21858117	(Quaranta *et al.*, 2011)
16	Nef DOWNREGULATES BCL2	15858021	(Priceputu *et al.*, 2005)
17	Nef DOWNREGULATES CCL3	20015995	(Nobile *et al.*, 2010)
18	Nef UPREGULATES IL12B	19019824	(Ma *et al.*, 2009)
19	Nef UPREGULATES IL6	11519483,8799208	(Smith *et al.*, 2001; Fiala *et al.*, 1996)
20	matrix UPREGULATES IL10	18178611	(Di Sabatino *et al.*, 2008)
21	matrix UPREGULATES IL1B	18593760	(Fan *et al.*, 2009)
22	matrix DOWNREGULATES IL2	21482826	(De Francesco *et al.*, 2011)
23	env_gp120 ACTIVATES CASP3	16330530	(Garg and Blumenthal, 2006)
24	env_ gp120 DOWNREGULATES CD4	22226668	(Toda *et al.*, 2011)
25	env_ gp160 UPREGULATES TNF	8938574	(Reuben *et al.*, 1996)
26	Vpu DOWNREGULATES BCL2	11696595	(Akari *et al.*, 2001)
27	env gp120 ACTIVATES MAPK8	11468147	(Misse *et al.*, 2001)
28	env gp120 INHIBITS TNF	16873189	(Buriani *et al.*, 1999)
29	Tat UPREGULATES NFKBIA	22187158	(Fiume *et al.*, 2012)
30	Tat UPREGULATES IFNB1	9223731	(Sanhadji *et al.*, 1997)
31	Tat INTERACTS WITH CXCR4	11594685	(De Clercq and Schols, 2001)
32	Tat UPREGULATES CCL3	15204927	(D'Aversa *et al.*, 2004)
33	Tat ACTIVATES CASP9	11509621	(Park *et al.*, 2001)
34	Nef INTERACTS WITH NFKB1	12419805	(Varin *et al.*, 2003)
35	Nef DOWNREGULATES CCL3	12370346	(Messmer *et al.*, 2002)
36	Nef DOWNREGULATES BCL2L1	11123279	(Rasola *et al.*, 2001)
37	Vpr ACTIVATES CASP9	12096338	(Muthumani *et al.*, 2002)
39	Vpr INDUCES RELEASE OF CYCS	16511342	(Moon and Yang, 2006)

Parent, M., Yung, T. M., Rancourt, A., Ho, E. L., Vispe, S., Suzuki-Matsuda, F., Uehara, A., Wada, T., Handa, H., and Satoh, M. S. (2005). Poly(ADP-ribose) polymerase-1 is a negative regulator of HIV-1 transcription through competitive binding to TAR RNA with Tat.positive transcription elongation factor b (p-TEFb) complex. *J. Biol. Chem.*, **280**(1), 448–457.

Park, I. W., Ullrich, C. K., Schoenberger, E., Ganju, R. K., and Groopman, J. E. (2001). HIV-1 Tat induces microvascular endothelial apoptosis through caspase activation. *J. Immunol.*, **167**(5), 2766–2771.

Perfettini, J. L., Nardacci, R., Seror, C., Bourouba, M., Subra, F., Gros, L., Manic, G., Amendola, A., Masdehors, P., Rosselli, F., Ojcius, D. M., Auclair, C., de The, H., Gougeon, M. L., Piacentini, M., and Kroemer, G. (2009). The tumor suppressor protein PML controls apoptosis induced by the HIV-1 envelope. *Cell Death Differ.*, **16**(2), 298–311.

Priceputu, E., Rodrigue, I., Chrobak, P., Poudrier, J., Mak, T. W., Hanna, Z., Hu, C., Kay, D. G., and Jolicoeur, P. (2005). The Nef-mediated AIDS-like disease of CD4C/human immunodeficiency virus transgenic mice is associated with increased Fas/FasL expression on T cells and T-cell death but is not prevented in Fas-, FasL-, tumor necrosis factor receptor 1-, or interleukin-1beta-converting enzyme-deficient or Bcl2-expressing transgenic mice. *J. Virol.*, **79**(10), 6377–6391.

Quaranta, M. G., Vincentini, O., Felli, C., Spadaro, F., Silano, M., Moricoli, D., Giordani, L., and Viora, M. (2011). Exogenous HIV-1 Nef upsets the IFN--induced impairment of human intestinal epithelial integrity. *PLoS ONE*, **6**(8), e23442.

Ragheb, J. A., Bressler, P., Daucher, M., Chiang, L., Chuah, M. K., Vandendriessche, T., and Morgan, R. A. (1995). Analysis of trans-dominant mutants of the HIV type 1 Rev protein for their ability to inhibit Rev function, HIV type 1 replication, and their use as anti-HIV gene therapeutics. *AIDS Res. Hum. Retroviruses*, **11**(11), 1343–1353.

Rasola, A., Gramaglia, D., Boccaccio, C., and Comoglio, P. M. (2001). Apoptosis enhancement by the HIV-1 Nef protein. *J. Immunol.*, **166**(1), 81–88.

Reuben, J. M., Turpin, J. A., Lee, B. N., Doyle, M., Gonik, B., Jacobson, R., and Shearer, W. T. (1996). Induction of inflammatory cytokines in placental monocytes of gravidae infected with the human immunodeficiency virus type 1. *J. Interferon Cytokine Res.*, **16**(11), 963–971.

Sanhadji, K., Leissner, P., Firouzi, R., Pelloquin, F., Kehrli, L., Marigliano, M., Calenda, V., Ottmann, M., Tardy, J. C., Mehtali, M., and Touraine, J. L. (1997). Experimental gene therapy: the transfer of Tat-inducible interferon genes protects

Table 2. Predicted Regulatory Interactions with their supporting PUBMED id and references.

SL.No.	Predicted Regulatory Interactions	PUBMED ID	Reference
1	env_gp160 inhibited by CCL4	21118814	(Garcia-Perez *et al.*, 2011)
2	env_gp160 inhibited by CCL5	21118814	(Garcia-Perez *et al.*, 2011)
3	env_gp160 inhibited by HDAC6	16148047	(Valenzuela-Fernandez *et al.*, 2005)
4	Rev imported by IPO7	16704975	(Arnold *et al.*, 2006)
5	capsid imported by IPO7	20147401	(Atasheva *et al.*, 2010)
6	Rev inhibited by CD4	8573391	(Ragheb *et al.*, 1995)
7	matrix inhibited by NFKBIA	10722660	(Flory *et al.*, 2000)
8	Capsid interacts with APOBEC3G	17065315	(Kamada *et al.*, 2006)
9	Vif interacts with TP53	21071676	(Flory *et al.*, 2000)
10	RT interacts with CD4	22426469	(An *et al.*, 2012)
11	Vif phosphorylated by MAPK3	10074203	(Yang and Gabuzda, 1999)
12	Vif ubiquitinated by UBB	15781449	(Kobayashi *et al.*, 2005)
13	Vif ubiquitinated by UBD	18596088	(DeHart *et al.*, 2008)
14	Vif ubiquitinated by MAPK1	10074203	(Yang and Gabuzda, 1999)
15	Gag_Pr55 inhibited by IFNA16	11197304	(Miterski *et al.*, 1999)
16	Gag_Pr55 inhibited by IFNA7	8553538	(Agy *et al.*, 1995)
17	Tat interacts with CD4	12457987	(Gibellini *et al.*, 2002)
18	Tat interacts with PRKCQ	9446795	(Borgatti *et al.*, 1998)
19	Tat interacts with LCK	18854243	(Wolf *et al.*, 2008)
20	p6 phosphorylated by MAPK3	11773377	(Muller *et al.*, 2002)
21	p6 phosphorylated by MAPK1	15155723	(Hemonnot *et al.*, 2004)
22	Gag_Pr55 inhibited by IFNA2	8553538	(Agy *et al.*, 1995)
23	env_gp160 interacts with TP53	19023333	(Perfettini *et al.*, 2009)
24	Nef interacts with CD28	21819585	(Olivieri *et al.*, 2011)

human cells against HIV-1 challenge in vitro and in vivo in severe combined immunodeficient mice. *AIDS*, **11**(8), 977–986.

Sarmady, M., Dampier, W., and Tozeren, A. (2011). Sequence- and interactome-based prediction of viral protein hotspots targeting host proteins: a case study for HIV Nef. *PLoS ONE*, **6**(6), e20735.

Smith, D. G., Guillemin, G. J., Pemberton, L., Kerr, S., Nath, A., Smythe, G. A., and Brew, B. J. (2001). Quinolinic acid is produced by macrophages stimulated by platelet activating factor, Nef and Tat. *J. Neurovirol.*, **7**(1), 56–60.

Toda, T., Kuwahara, K., Kondo, N., Matsuda, Z., Maeda, Y., Maeda, K., and Sakaguchi, N. (2011). Dynamic appearance of antigenic epitopes effective for viral neutralization during membrane fusion initiated by interactions between HIV-1 envelope proteins and CD4/CXCR4. *Immunobiology*.

Trible, R. P., Emert-Sedlak, L., and Smithgall, T. E. (2006). HIV-1 Nef selectively activates Src family kinases Hck, Lyn, and c-Src through direct SH3 domain interaction. *J. Biol. Chem.*, **281**(37), 27029–27038.

Valenzuela-Fernandez, A., Alvarez, S., Gordon-Alonso, M., Barrero, M., Ursa, A., Cabrero, J. R., Fernandez, G., Naranjo-Suarez, S., Yanez-Mo, M., Serrador, J. M., Munoz-Fernandez, M. A., and Sanchez-Madrid, F. (2005). Histone deacetylase 6 regulates human immunodeficiency virus type 1 infection. *Mol. Biol. Cell*, **16**(11), 5445–5454.

Varin, A., Manna, S. K., Quivy, V., Decrion, A. Z., Van Lint, C., Herbein, G., and Aggarwal, B. B. (2003). Exogenous Nef protein activates NF-kappa B, AP-1, and c-Jun N-terminal kinase and stimulates HIV transcription in promonocytic cells. Role in AIDS pathogenesis. *J. Biol. Chem.*, **278**(4), 2219–2227.

Wolf, D., Witte, V., Clark, P., Blume, K., Lichtenheld, M. G., and Baur, A. S. (2008). HIV Nef enhances Tat-mediated viral transcription through a hnRNP-K-nucleated signaling complex. *Cell Host Microbe*, **4**(4), 398–408.

Wong, J. K., Campbell, G. R., and Spector, S. A. (2010). Differential induction of interleukin-10 in monocytes by HIV-1 clade B and clade C Tat proteins. *J. Biol. Chem.*, **285**(24), 18319–18325.

Yang, X. and Gabuzda, D. (1999). Regulation of human immunodeficiency virus type 1 infectivity by the ERK mitogen-activated protein kinase signaling pathway. *J. Virol.*, **73**(4), 3460–3466.

Zeng, Y., Zhang, X., Huang, Z., Cheng, L., Yao, S., Qin, D., Chen, X., Tang, Q., Lv, Z., Zhang, L., and Lu, C. (2007). Intracellular Tat of human immunodeficiency virus type 1 activates lytic cycle replication of Kaposi's sarcoma-associated herpesvirus: role of JAK/STAT signaling. *J. Virol.*, **81**(5), 2401–2417.

Zhao, T., Adams, M. H., Zou, S. P., El-Hage, N., Hauser, K. F., and Knapp, P. E. (2007). Silencing the PTEN gene is protective against neuronal death induced by human immunodeficiency virus type 1 Tat. *J. Neurovirol.*, **13**(2), 97–106.
